# Supplementary material for: Biomimetic caged platinum catalyst for hydrosilylation reaction with high site selectivity
Source: Nat Commun. 2021 Jan 4;12:64. doi: 10.1038/s41467-020-20233-w (PMC7782696; doi:10.1038/s41467-020-20233-w)
Supplement: Supplementary file 1 — Supplementary Information [file 41467_2020_20233_MOESM1_ESM.pdf]

Supplementary Information

Biomimetic Caged Platinum Catalyst for Hydrosilylation Reaction with High Site Selectivity

Ganghuo Pan[1], Chunhua Hu[2], Song Hong[3], Huaping Li[1], Dongdong Yu[1], Chengqian Cui[1], Qiaosheng Li[1], Nianjie Liang[1], Ying Jiang[1], Lirong Zheng[5], Lei Jiang[1], Yuzhou Liu[1,4*]

1. School of Chemistry, Beihang University, Beijing, 100191, PR China
2. The Department of Chemistry, New York University, 100 Washington Square East, New York, New York, 10003-6688, USA
3. Center for Instrumental Analysis, Beijing University of Chemical Technology, Chaoyang, Beijing 100029, China
4. Beijing Advanced Innovation Center for Biomedical Engineering, Beihang University, Beijing 100191, PR China
5. Beijing Synchrotron Radiation Facility, Institute of High Energy Physics, Chinese Academy of Sciences, Beijing 100049, P.R. China

*Correspondence to: liuyuzhou@buaa.edu.cn

Supplementary Section 1 Synthesis

1-1：Synthesis of hexaphenylbenzene (**HPB**) [1].

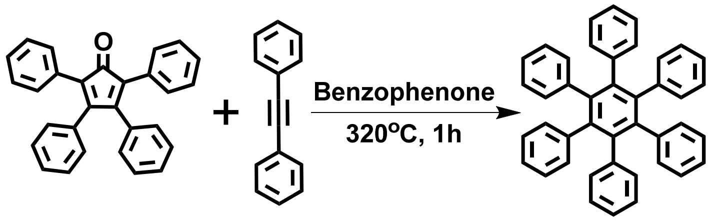

HPB

Supplementary Figure 1: Synthesis of **HPB**

A mixture of tetraphenylcyclopentadienone (8.0 g, 21.0 mmol), diphenylacetylene (8.0 g, 43.0 mmol), and benzophenone (40 g) was heated at 320 °C

for 1 h in a one-neck round-bottom flask attached to an air condenser. The reaction mixture was cooled to room temperature to give a crystalline precipitate, which was filtered, washed with benzene, and dried in a vacuum oven at 80 °C overnight. The product was purified further by recrystallization from diphenyl ether, filtered, and washed with hot benzene to give 8.1 g **HPB** (72%). ^{1}H NMR (300 MHz, $CDCl_3$) δ 6. 84 (br, 30H), HRMS (ESI TOF, positive) m/z calcd. for $C_{42}H_{31}$ $[M+H]^+$: 535.24258, found: 535.23772.

1-2: Synthesis of hexakis(4-bromophenyl) benzene (**HBB**)[2].

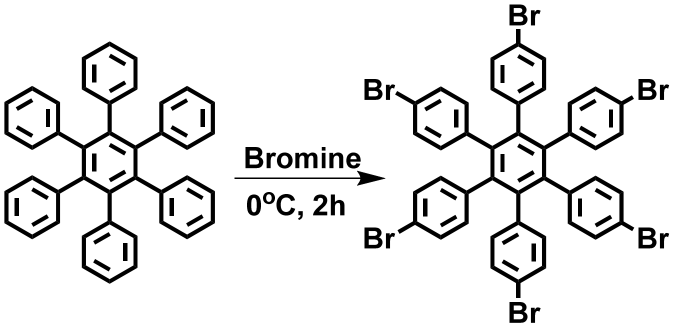

HPB **HBB**

Supplementary Figure 2: Synthesis of **HBB**

After dropwise addition of bromine (400.0 mL, 8 mol) into hexaphenylbenzene (100 g, 187 mmol) at 0 ˚C, the mixture was vigorously stirred for 2 h at rt, and then poured into ice-cooled aqueous solution of Na_2SO_3. After stirring overnight, the resulting precipitation was filtered and washed with water, acetone, and CH_2Cl_2 successively to afford **HBB** (153 g, 81%) as a white solid. Refer to Supplementary Fig. 6 for ^{1}H-NMR spectrum and Supplementary Fig. 7 for MALDI-TOF-MS spectrum. ^{1}H NMR (400 MHz, $CDCl_3$) δ 7.07-7.04 (d, 12H), 6.62–6.60 (d, 12H); MALDI-TOF-MS m/z calcd. for $C_{42}H_{24}Br_6$ $[M]^+$: 1007.6917, found:1007.7993.

1-3: Synthesis of **TBTO** [3].

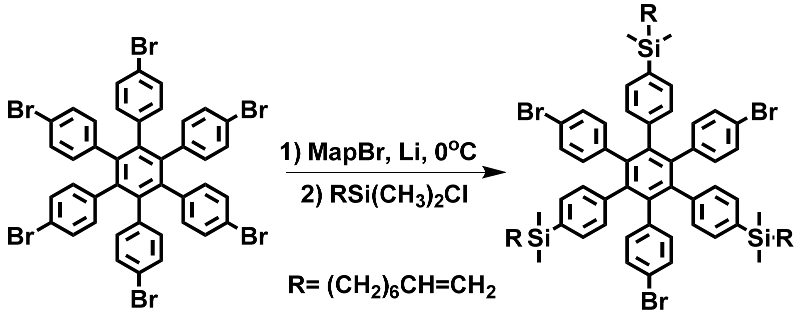

HBB **TBTO**

Supplementary Figure 3: Synthesis of **TBTO**

To granular lithium (1.86 g, 0.268 mol) was added the solution of *p*-(dimethylamino)benzene (MapBr) (26.8 g, 0.134 mol) in Et_2O (100 mL) dropwise in 10 min. During the addition, the solution spontaneously refluxed. After the addition, the mixture was refluxed for 1 h. Then THF (200 mL) and compound **HBB** (30.0 g, 29.8 mmol) were added at 0 °C. The mixture was stirred for 20 min at 0 °C and transferred to another reaction vessel containing $CH_2=CH(CH_2)_6Si(CH_3)_2Cl$ (54.8g 0.268 mol) at 0 °C, and then the solvent was removed in vacuum. After the addition of water (100 mL) and $CHCl_3$ (100 mL), the organic layer was separated. The aqueous layer was extracted with $CHCl_3$ (100 mL×3) and the combined extracts were washed twice with aq. HCl (1 M). The resulting organic solution was dried over anhydrous $MgSO_4$, and filtered. Then the solvent was removed in vacuum and the product was purified by flash chromatography on silica gel with dichloromethane/hexanes (2:8) mixture as the eluent to afford product of **TBTO** as a white solid 28.1g (74%). Refer to Supplementary Fig. 8 for NMR spectrum. ^1H NMR (400 MHz, $CDCl_3$) δ 0.13 (s, 18H), 0.60-0.54 (t, 6H), 1.35-1.12 (m, 24H), 2.07-2.00 (q, 6H), 5.02-4.91 (m, 6H), 5.88-5.75 (m, 3H),6.64-6.61 (d, 6H), 6.73-6.70 (d, 6H), 6.95-6.93 (d, 6H), 7.03-7.00 (d, 6H); MALDI-TOF-MS m/z calcd. for $C_{72}H_{88}Br_3Si_3$ $[M+H]^+$:1275.37235, found:1275.37641.

1-4: Synthesis of **H3L8-Me**[4].

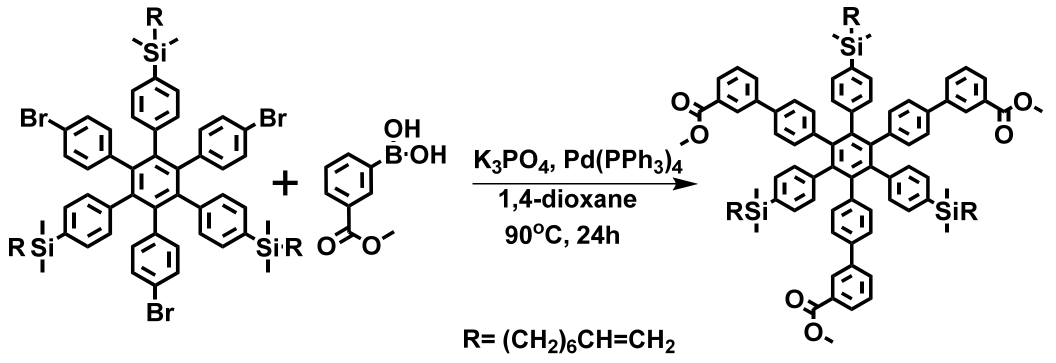

TBTO **H3L8-Me**

Supplementary Figure 4: Synthesis of **H3L8-Me**

TBTO（2 g, 1.5mmol）, benzene-3-carboxyethylesterboronic acid (0.65 g, 5 mmol), and K_3PO_4 (2.0 g, 10.0 mmol) were added to 1,4-dioxane (50 mL), and the mixture was de-aerated under N_2. $Pd(PPh_3)_4$ (0.1 g, 0.08 mmol) was added to the reaction mixture with stirring, and then the mixture heated at 90℃ for 24h under N_2. The resultant mixture was evaporated to dryness and taken up in $CHCl_3$ which had been dried over $MgSO_4$. The $CHCl_3$ solution was evaporated to dryness and the residue was purified by silica gel column chromatography, to give **H3L8-Me** 1.9g (85%). Refer to Supplementary Fig. 9 for NMR spectrum. ^1H NMR (400 MHz, $CDCl_3$) δ 0.07 (s, 18H), 0.53-0.51 (br, 6H), 1.25-1.07 (m, 24H), 1.95-1.88 (q, 6H), 3.90 (s, 9H), 4.97-4.88 (m, 6H), 5.82-5.68 (m, 3H), 6.86-6.84 (d, 6H), 6.92-6.89 (d, 6H) , 7.02-6.99 (d, 6H), 7.14-7.11 (d, 6H), 7.42-7.37 (t, 3H), 7.60-7.57 (d, 3H) , 7.94-7.91 (d, 3H), 8.09 (s, 3H); HRMS (ESI TOF, positive) m/z calcd. for $C_{96}H_{108}O_6Si_3Na$ $[M+Na]^+$: 1464.73850, found: 1464.73581.

1-5: Synthesis of **L1**

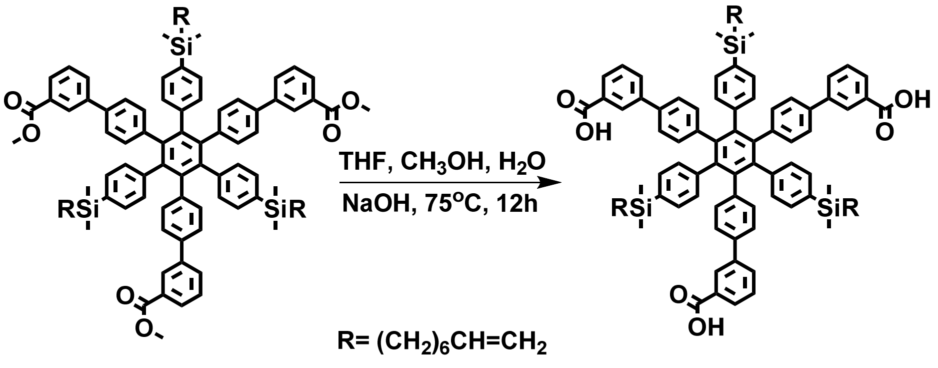

H3L8-Me **L1**

Supplementary Figure 5: Synthesis of **L1**

H3L8-Me (1.9 g) was hydrolyzed by refluxing in the solution of 2 M aqueous NaOH(10 ml)/ THF (10 ml)/ CH_3OH (10 ml) for about 12h followed by acidification with 37% HCl to afford **L1**. The crude product was recrystallized from DMF/H_2O. Refer to Supplementary Fig. 10 for ^{1}H NMR spectrum and Supplementary Fig. 11 for ^{13}C NMR spectrum. ^{1}H NMR (400 MHz, DMSO-d6) δ 0.00 (s, 6H), 0.40-0.36 (br, 6H), 1.06-0.89 (m, 24H), 1.85-1.78 (q, 6H), 4.95-4.86 (m, 6H), 5.76-5.63 (m, 3H), 7.02-6.93 (m, 18H), 7.13-7.11 (d, 6H), 7.26-7.21 (t, 3H), 7.38-7.35 (d, 3H) , 7.76-7.74 (d, 3H), 7.95 (s, 3H), 13.24(br,3H); ^{13}C NMR (100MHz, DMSO-d6) δ 169.34, 143.44, 142.76, 142.24, 141.98, 141.80, 141.52, 139.61, 137.92, 134.34, 134.16, 133.09, 130.58, 130.38, 129.83, 127.31, 116.94, 35.96, 35.14, 30.91, 30.71, 25.76, 20.44, 18.01, 0.50; ^{29}Si NMR (120MHz, THF) δ -3.114. HRMS (ESI TOF, negative) m/z calcd. for $C_{93}H_{101}O_6Si_3$ $[M-H]^-$: 1398.69450, found: 1398.68861. Element analysis (EA): C, 79.53; H, 7.21 (calcd.: C, 79.78; H, 7.34).

Supplementary Section 2: Tables and Figures

Empirical formula	$C_{780} H_{876} Cu_{12} N_{12} O_{60}Si_{24}$
Formula weight	12903.02
Temperature	100 K
Wavelength	0.60000 Å
Crystal system	trigonal
Space group	R -3
Unit cell dimensions	a = 47.215(7) Å α = 90°.<br>b = 47.215(7) Å β = 90°.<br>c = 68.829(14) Å γ = 120°
Volume	132879(46) Å^3
Z	6
Density	0.961 g/cm3
Crystal size	0.3 x 0.27x 0.1 mm^3
Theta range for data collection	1.518 to 25.181°.
Index [h,k,l]max	56,49,81
Reflections collected	52407
Independent reflections	79108 [R(int) = 0.0259]
Completeness to theta = 25.181°	99.6%
Absorption correction	Empirical
Refinement method	Full-matrix least-squares on F2
Goodness-of-fit on F2	1.327
Final R indices [I>2sigma(I)]	R_1 = 0.1116, wR_2 = 0.3071
R indices (all data)	R_1 = 0.0991, wR_2 = 0.2868

Supplementary Table 1. Crystal data and structure refinement data for **MOP1**.

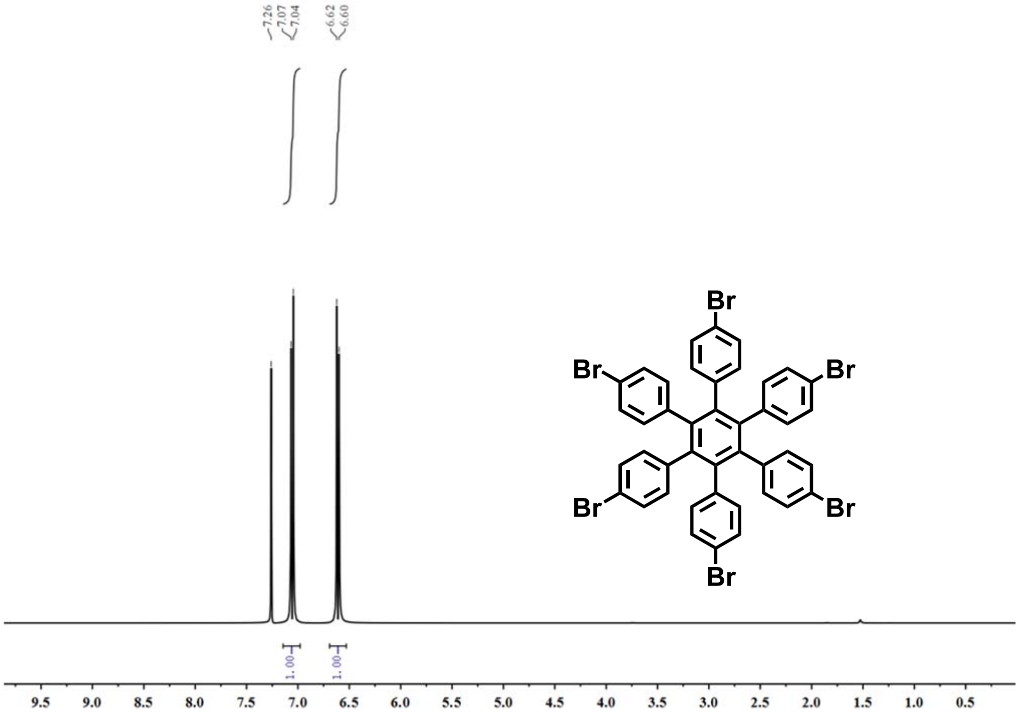

Supplementary Figure 6. ^{1}H-NMR of **HBB**

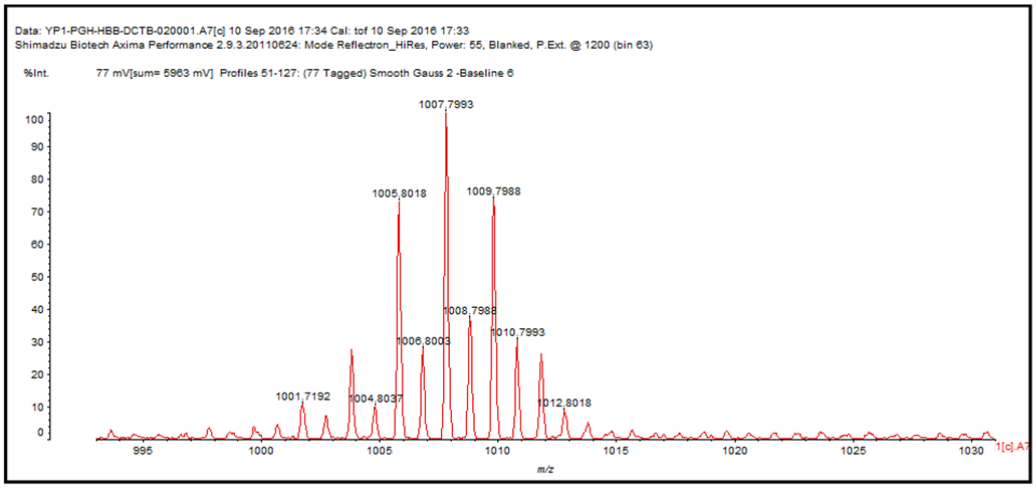

Supplementary Figure 7. MALDI-TOF-MS of **HBB**

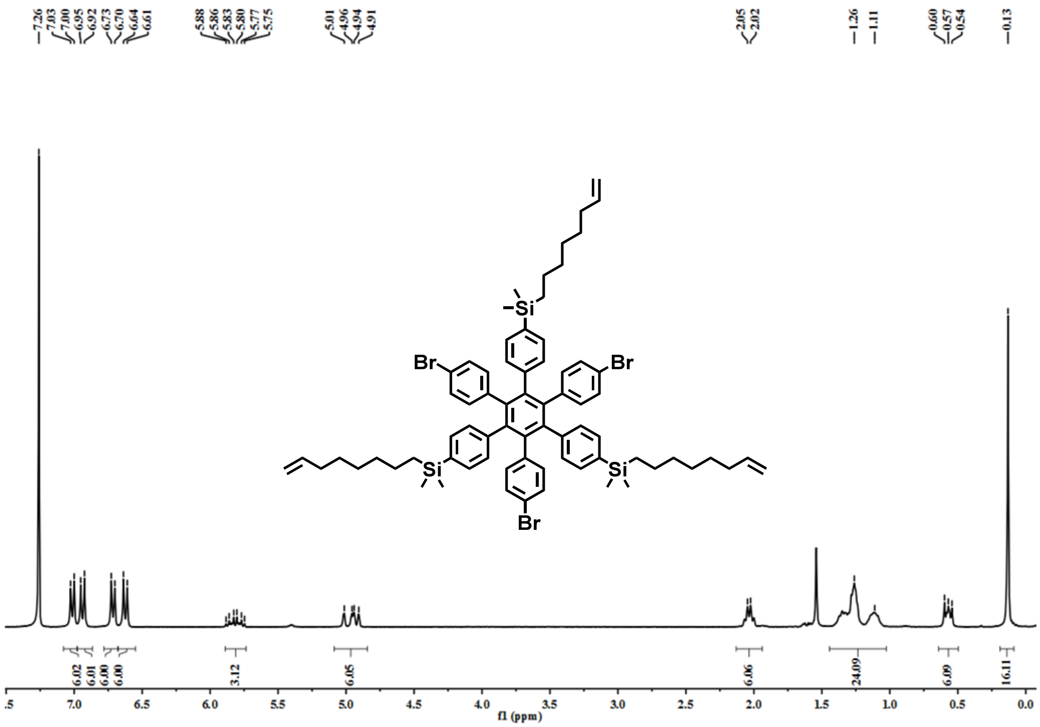

Supplementary Figure 8. ^{1}H-NMR of **TBTSi-O**

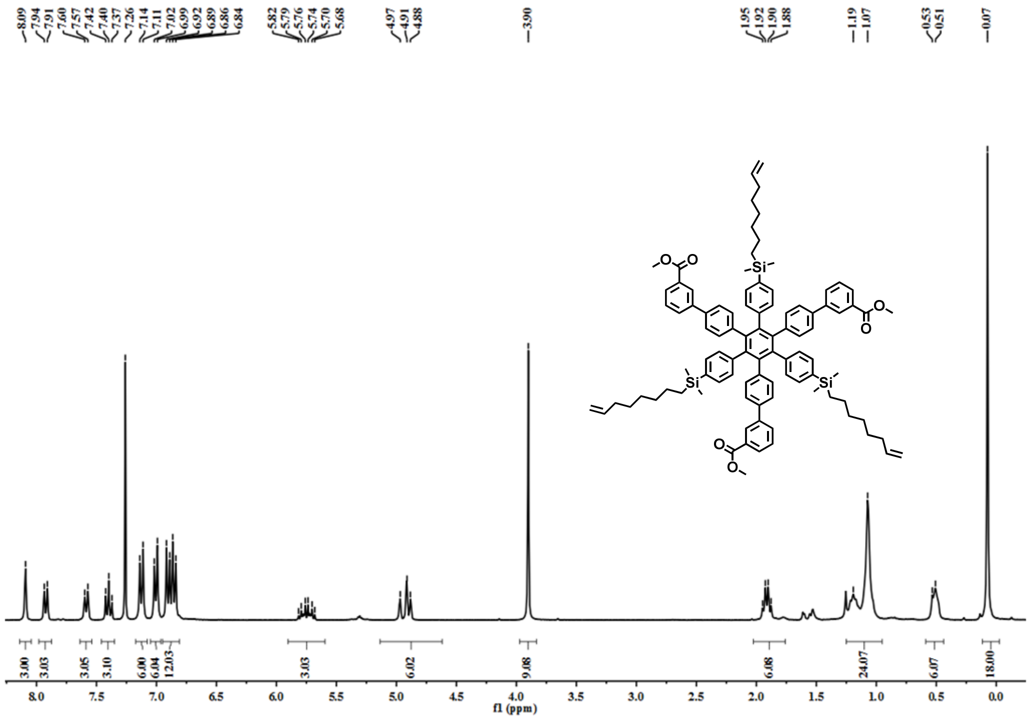

Supplementary Figure 9. ^{1}H-NMR of **H3L8-Me**

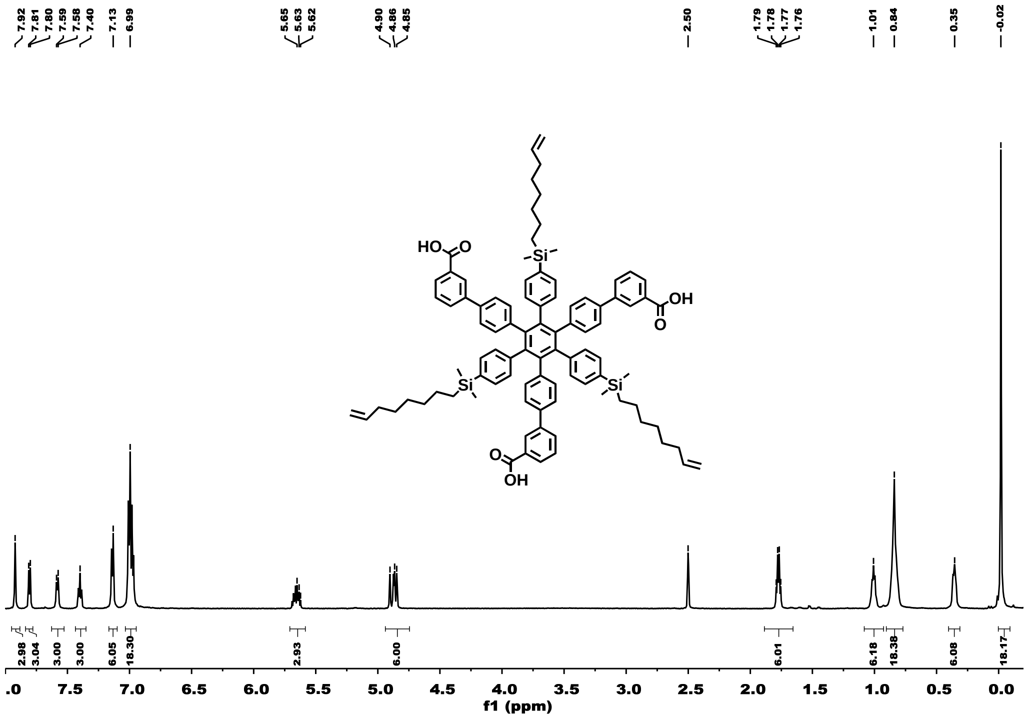

Supplementary Figure 10. ^{1}H-NMR of **L1**

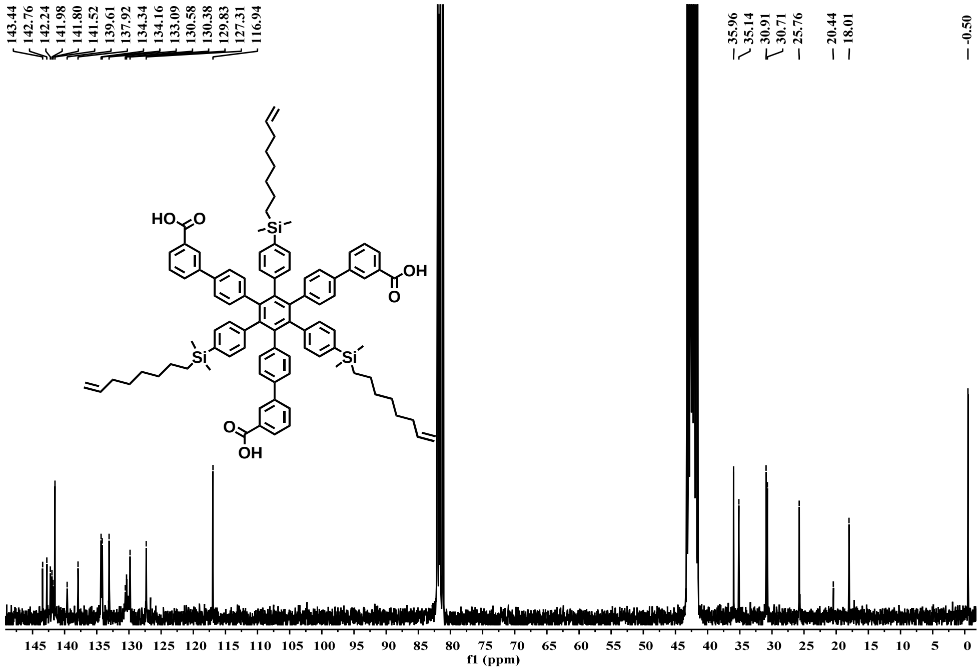

Supplementary Figure 11. ^{13}C-NMR of **L1**

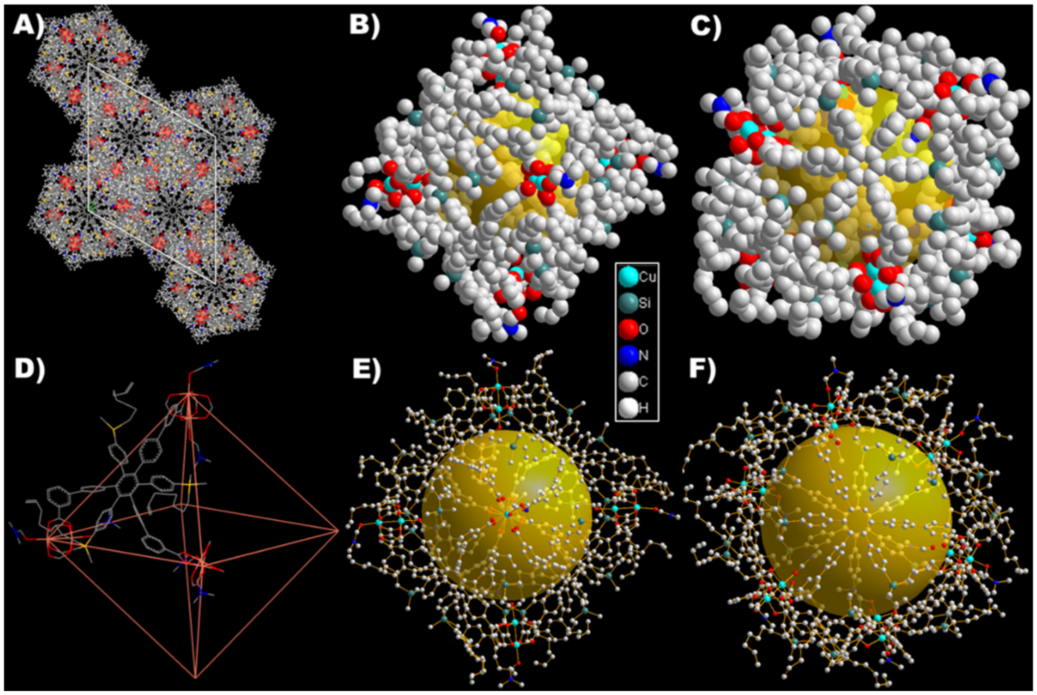

Supplementary Figure 12. A) The unit cell shown along the Z axis direction; B, C) Single-crystal structure of **MOP1** in space-filling model; D) The schematic drawing of the cage showing an octahedron-shape, which is similar to the one reported by Shionoya group (*J. Am. Chem. Soc.* **131**, 11646−11647 (2009))[5] and Lah group (*Inorg. Chem.* **48**, 1281−1283 (2009))[6]; E, F) The corresponding ball and stick model to B and C. The hydrogen atoms are deleted for clarity.

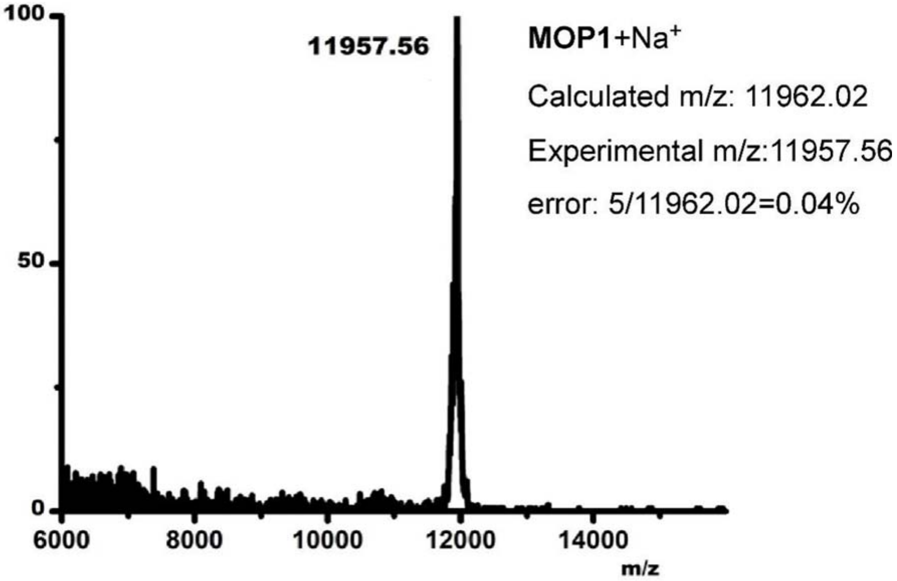

Supplementary Figure 13. MALDI-TOF-MS of **MOP1** with comparison between experimental and calculated values

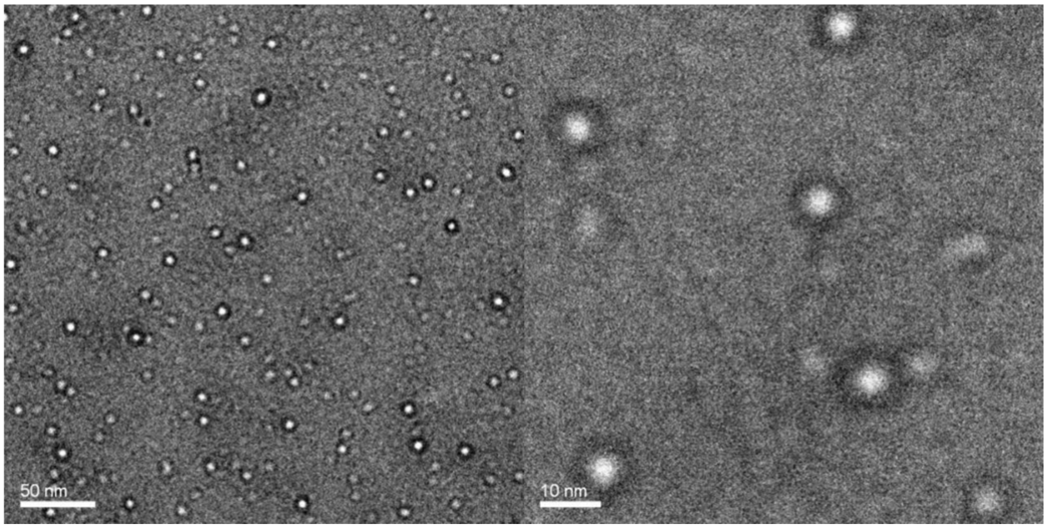

Supplementary Figure 14. HR-TEM of **MOP1** deposited from its DMF solution on the carbon support. The sizes are uniformly around 5 nm, consistent with the relative size estimation based on its single-crystal structure.

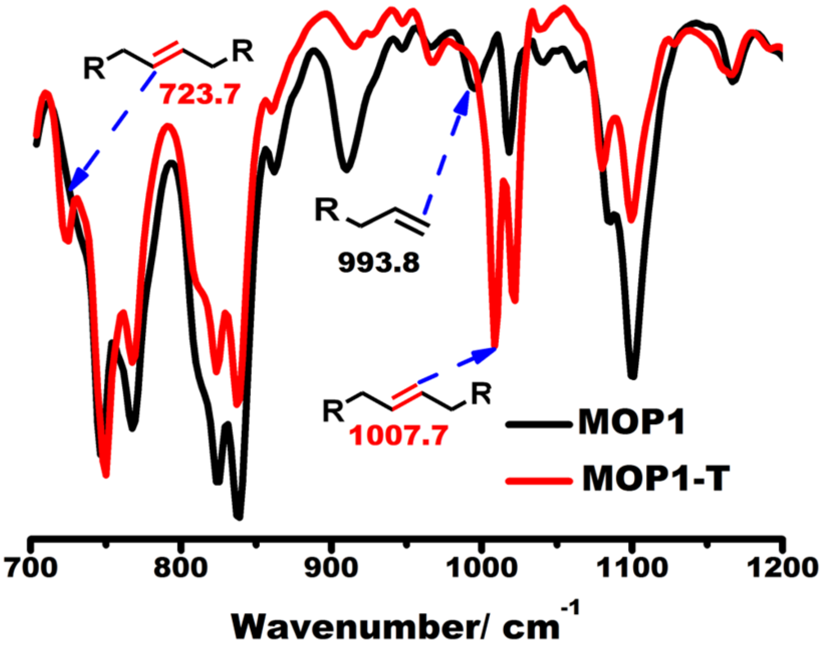

Supplementary Figure 15. IR of the **MOP1** compared with that from **MOP1-T**, showing the full disappearance of terminal alkene groups with appearance of internal alkene groups in **MOP1-T**.

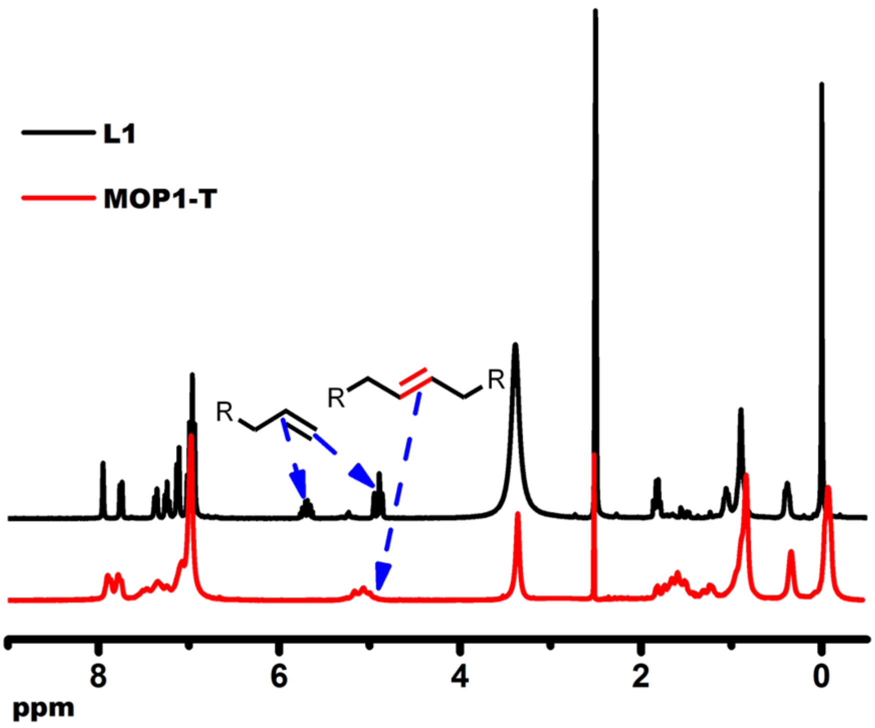

Supplementary Figure 16. Comparison between ^{1}H-NMR spectra of **L1** and **MOP1-T**, showing the successful and full metathesis transformation.

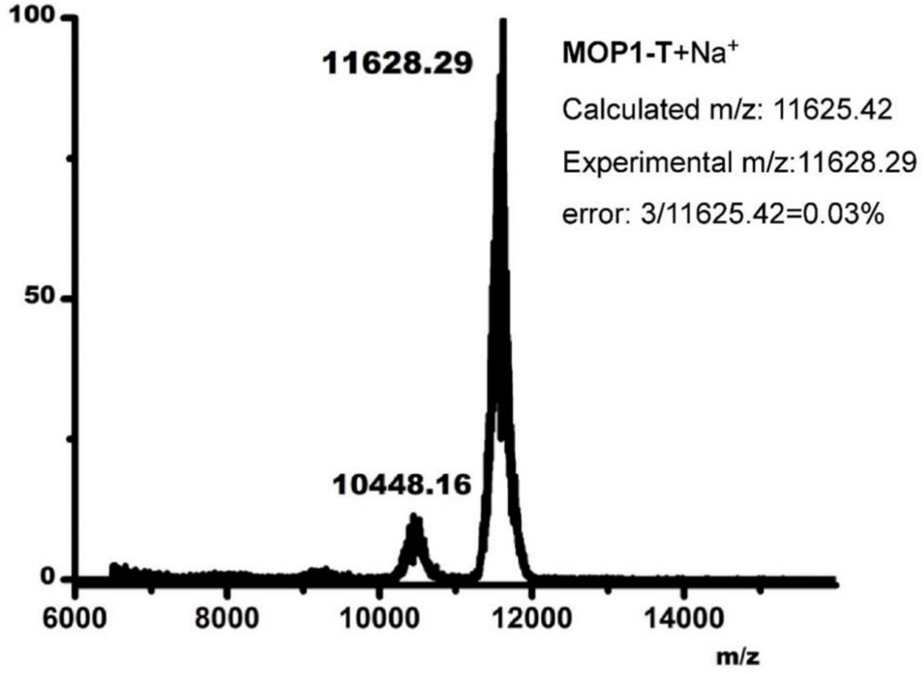

Supplementary Figure 17. MALDI-TOF-MS of **MOP1-T** with comparison between experimental and calculated values. The peak at 10448 (m/z) is related to **MOP1-T** with the loss of some unknown species during reaction or ionization process.

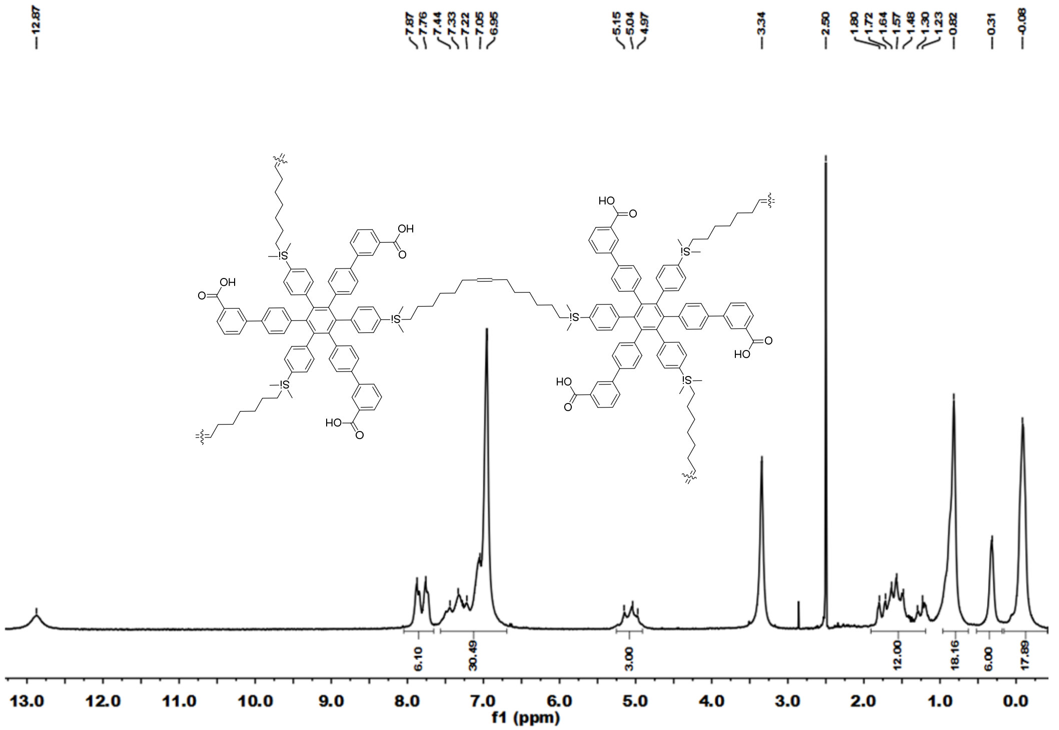

Supplementary Figure 18. ^{1}H-NMR of **COP1-T**

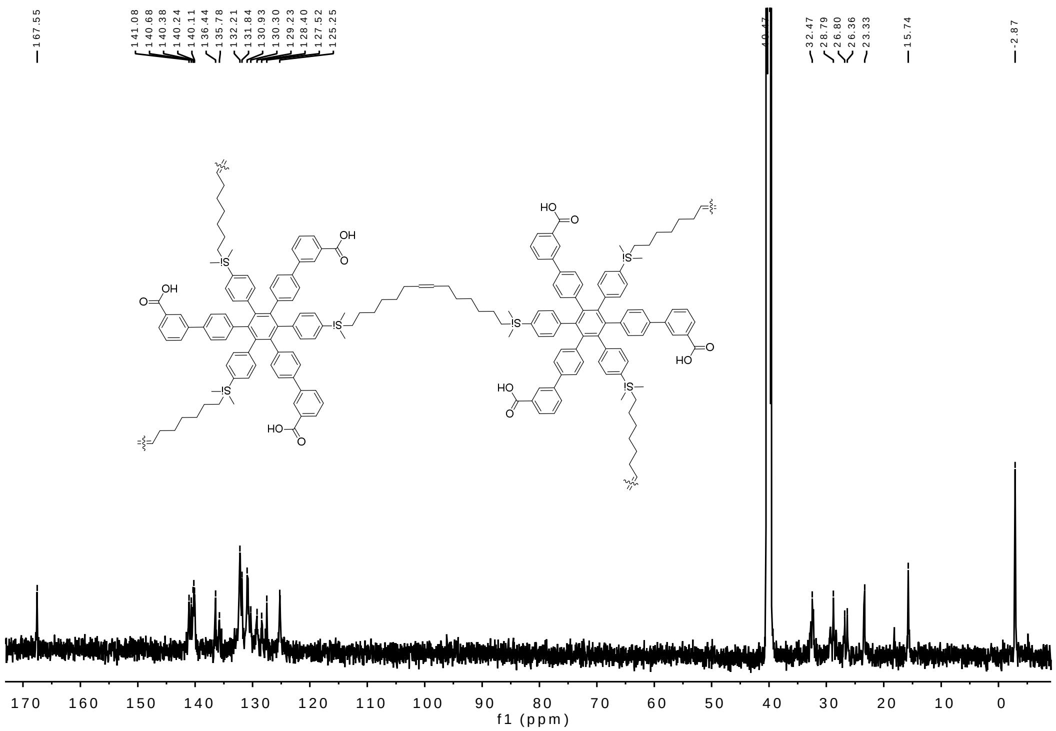

Supplementary Figure 19. ^{13}C-NMR of **COP1-T**

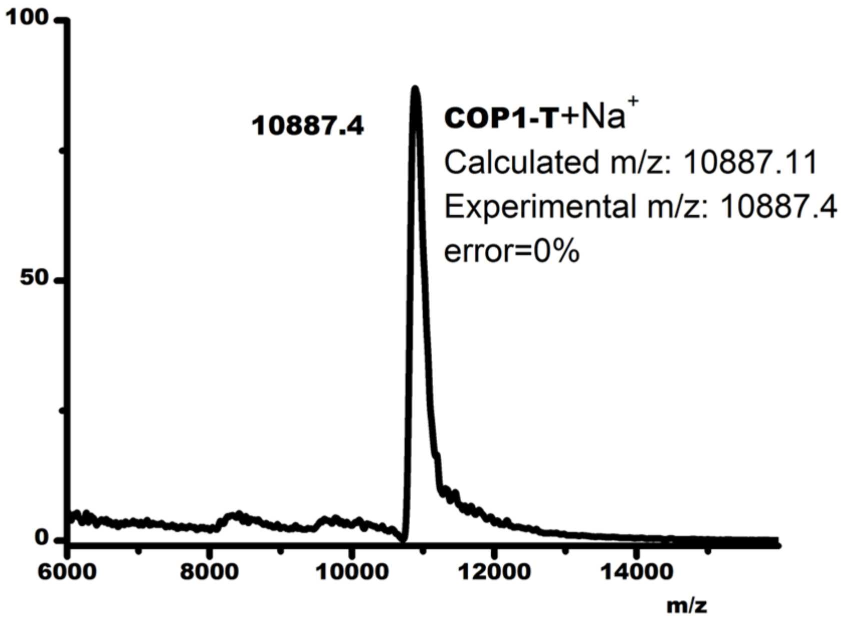

Supplementary Figure 20. MALDI-TOF-MS of the **COP1-T** with comparison between experimental and calculated values.

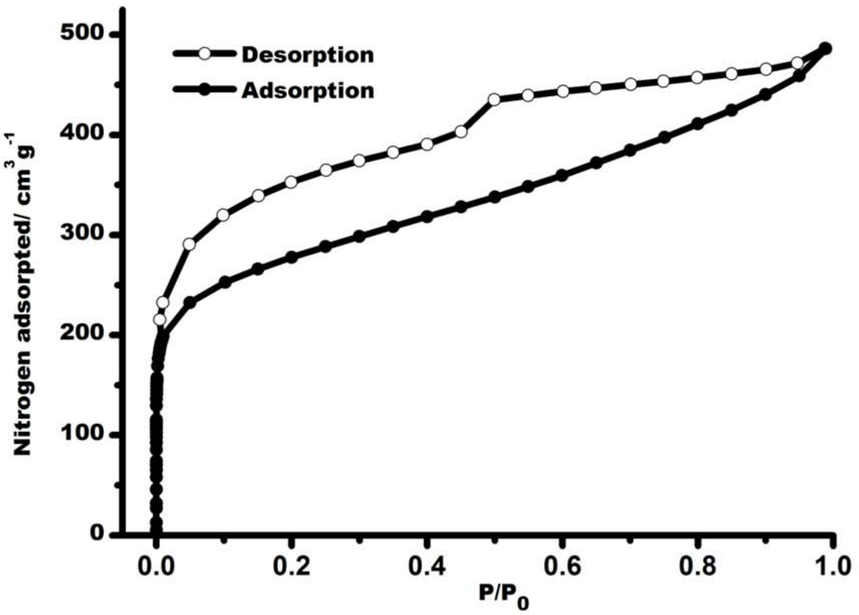

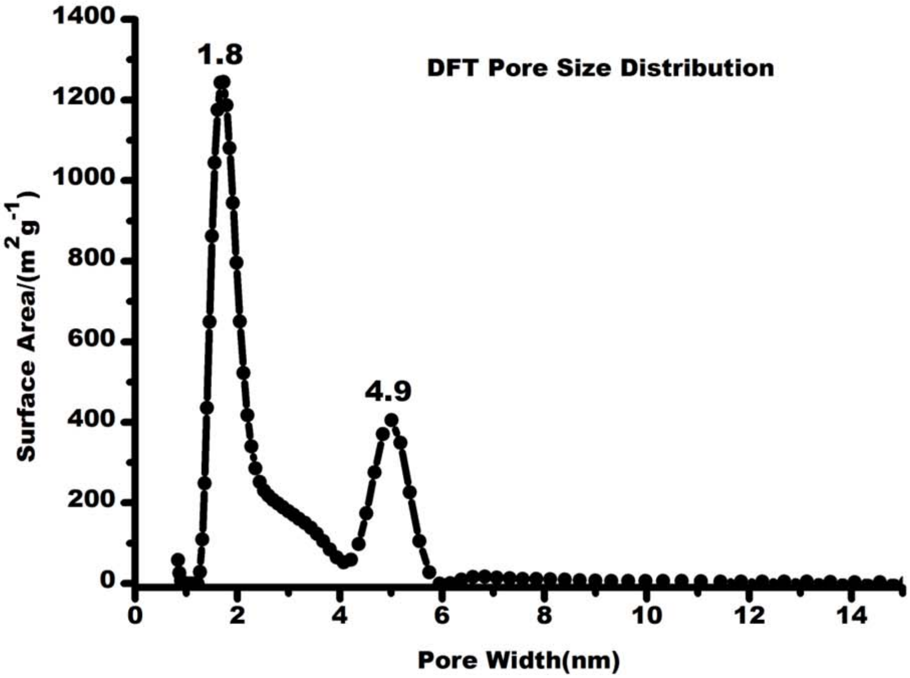

Supplementary Figure 21. Nitrogen sorption isotherms for **COP1-T** at 77 K and relative pore size distribution. Estimated total BET surface was around 999.9 m^2/g based on the QSDFT model. The 1.8 nm pore width correspond to the free volume within the cage of **COP1-T** while 4.9 nm peak probably is related to the inter-cage space. Pore size and volume for **MOP1** were estimated based on its crystal structure using the software of Zeo++[7].

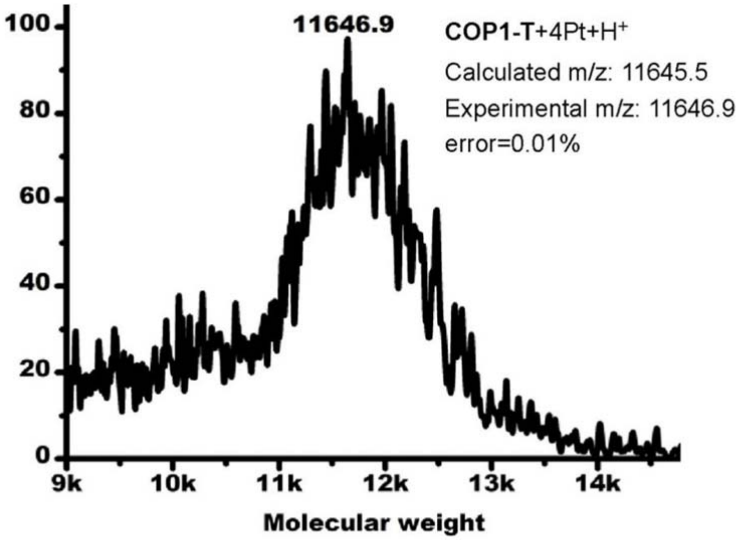

Supplementary Figure 22. MALDI-TOF-MS of the **COP1-T-Pt**. The averaged incorporated Pt atoms per **COP1-T** cage was estimated to be 3.89 based on the comparison between this spectrum and that of **COP1-T**. The broad peak width was characteristic for cages with trapped Pt atoms. For example, Chen et al. reported the MALDI-TOF characterization of calixarene cage with trapped Pt_6, Pt_{12} and Pt_{18} clusters, and similar broad peak widths were found (*J. Am. Chem. Soc.* **138**, 16236−16239 (2016))[8]. The broad peak width, especially the tail in high MW side, might be related to the trapped solvent molecules such as THF and water, which were evidently revealed in TGA (Supplementary Fig. 27, thermogravimetric analysis) of **COP1-T-Pt**.

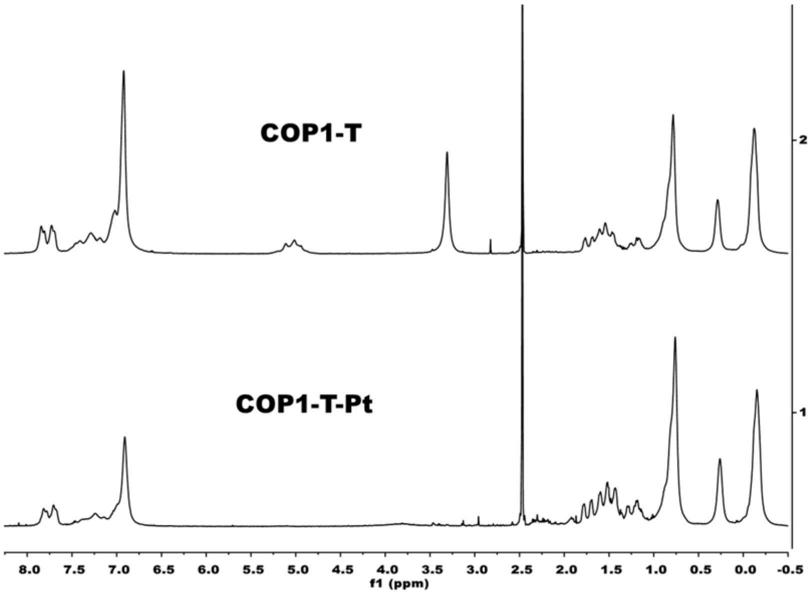

Supplementary Figure 23. ^{1}H-NMR spectra of **COP1-T** and **COP1-T-Pt**. The up-field shifting of the internal alkene hydrogen atoms from around 5.0 ppm to 2.0

ppm is a direct evidence for alkene-Pt coordination (*Coordin. Chem. Rev.* ***133***, 67−114 (1994))[9].

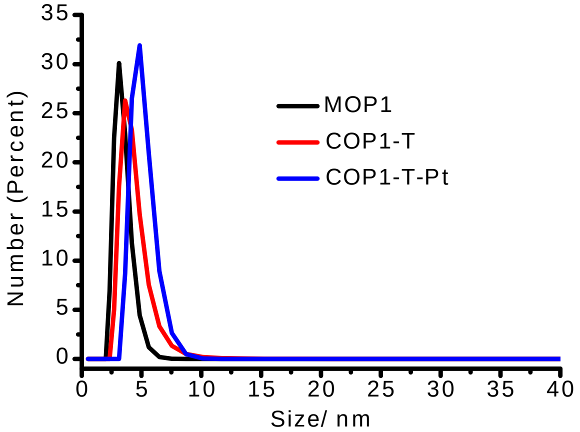

Supplementary Figure 24. Particle size distribution of **MOP1** (3.1 nm), **COP1-T** (3.6 nm) and **COP1-T-Pt** (4.8 nm) estimated by DLS in THF (~0.01M). The expanded size of **COP1-T** compared to **MOP1** is consistent with the successful tying through the long alkyl chain after metathesis reaction. The even large size of **COP1-T-Pt** is not clear, but probably is due to increased interaction between THF solvent molecules with trapped Pt atoms, which subsequently leads to an enhanced hydrodynamic radius. However, the size of **COP1-T-Pt** shows that it is definitely not an aggregate of multiple cages.

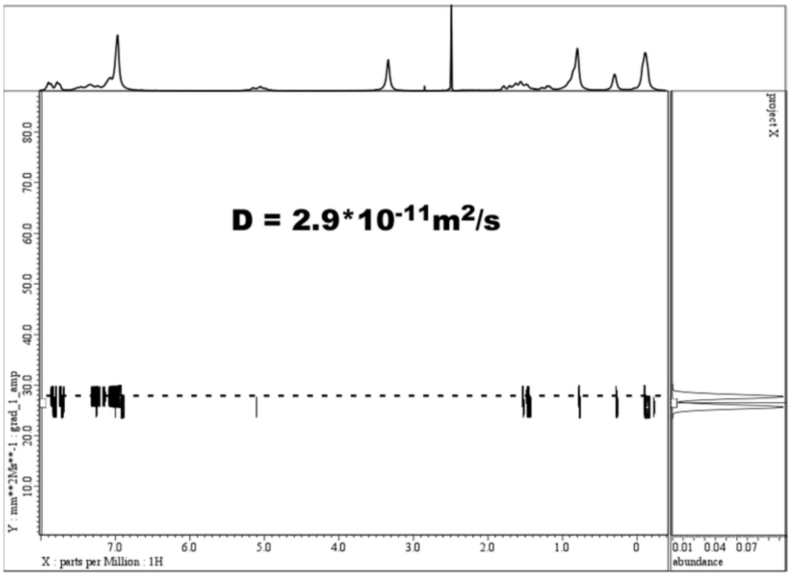

Supplementary Figure 25. ^{1}H-DOSY spectrum (600 MHz, DMSO-d6, 273 K) of **COP1-T**.

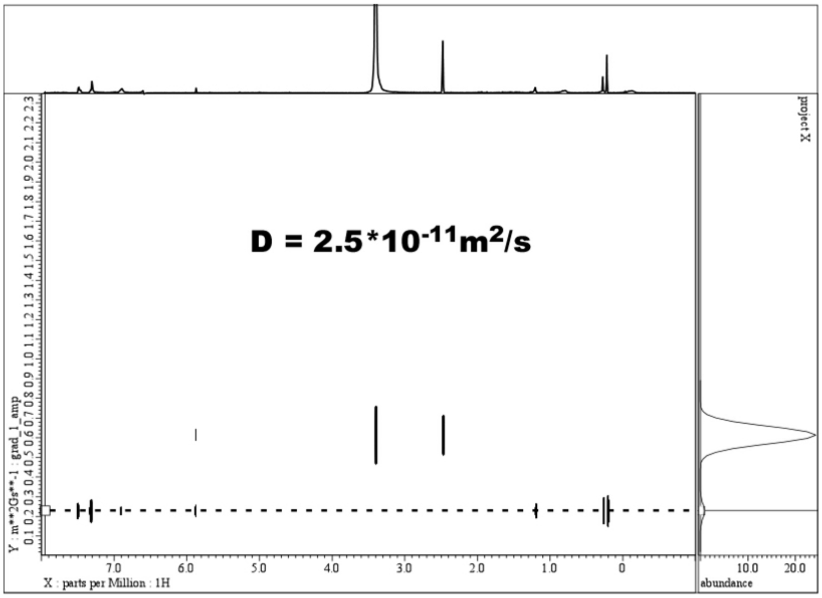

Supplementary Figure 26. ^{1}H-DOSY spectrum (600 MHz, DMSO-d6, 273 K) of **COP1-T-Pt**. Its similar diffusion coefficient as that of **COP1-T** is an indication of absence of cage aggregation during the introduction of Pt ions into the cage.

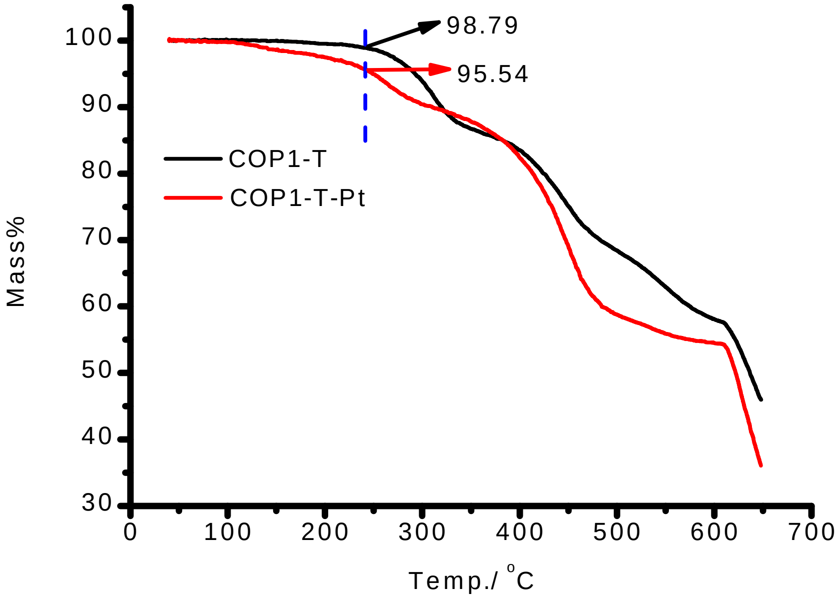

Supplementary Figure 27. TGA of **COP1-T** and **COP1-T-Pt** in argon with the heating rate of 10°C/min. The around 4.3% difference between **COP1-T** and **COP1-T-Pt** was attributed to trapped water and/or THF molecules within the cage for **COP1-T-Pt**, probably through Pt-O coordination.

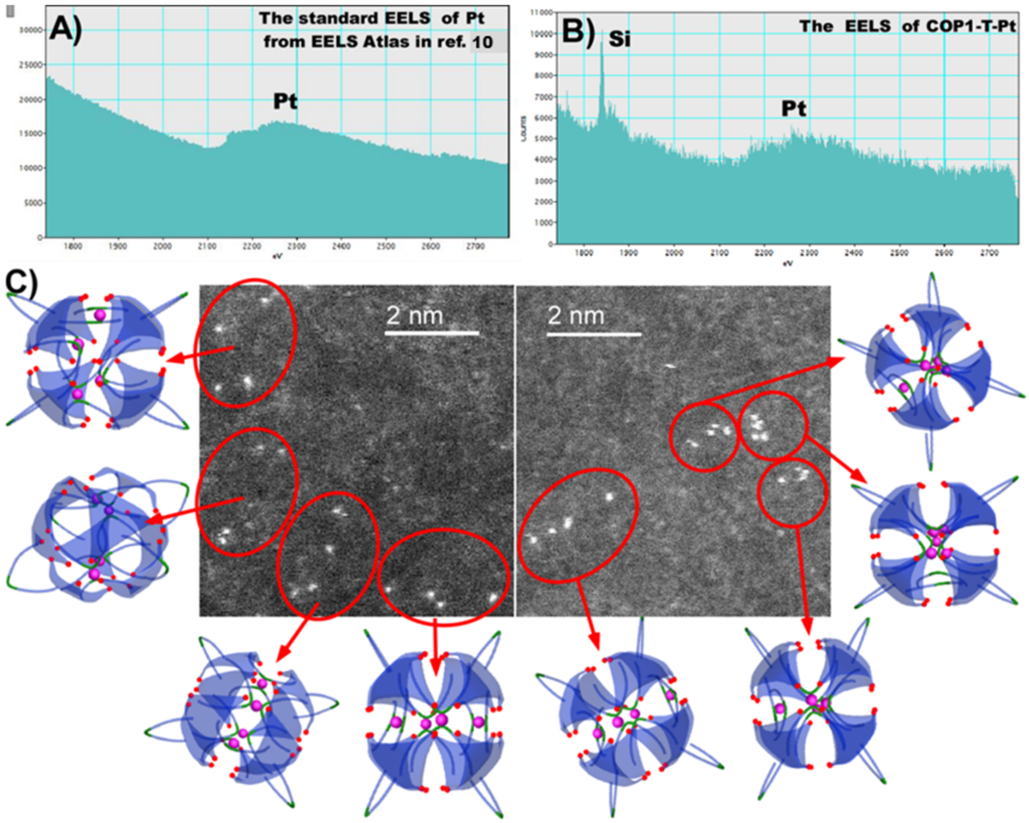

Supplementary Figure 28. (B) Electron Energy Loss Spectroscopy (EELS) for the ACHAADF- STEM image revealing the presence of Pt atoms with the comparison with standard EELS of Pt from EELS Atlas in ref. 10 (A). (C) ACHAADF-STEM images of **COP1-T-Pt** equipped with 3D counterpart of conformation, revealing the presence of single Pt atoms.

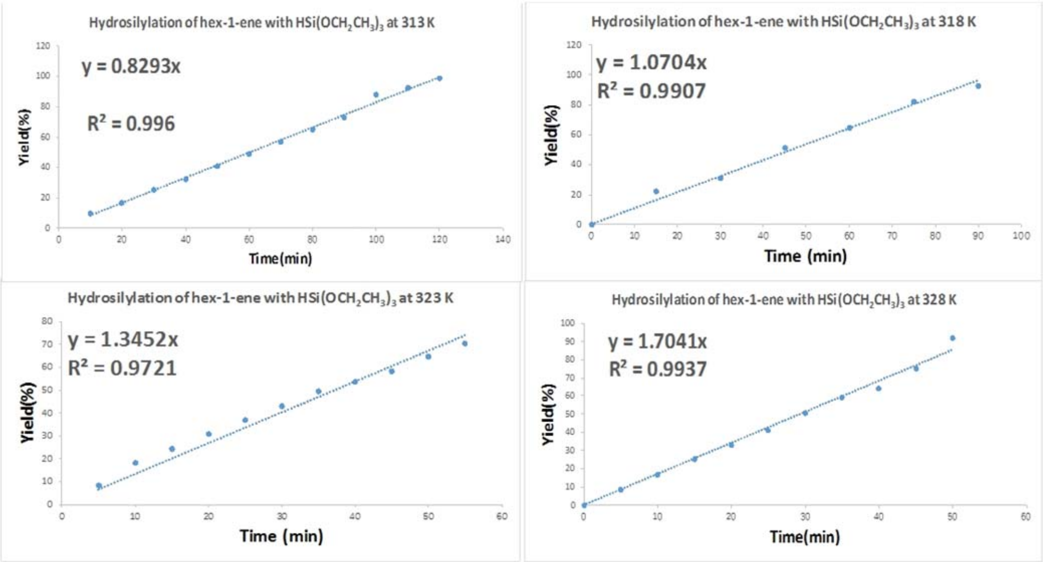

K	k	1/(Tc*R)	lnk	Ea(kJ/mol)
313.15	0.8293	0.000384094	-0.187173	40.827
318.15	1.0704	0.000378058	0.0680324	
323.15	1.3452	0.000372208	0.2965427	
328.15	1.7041	0.000366537	0.5330371	

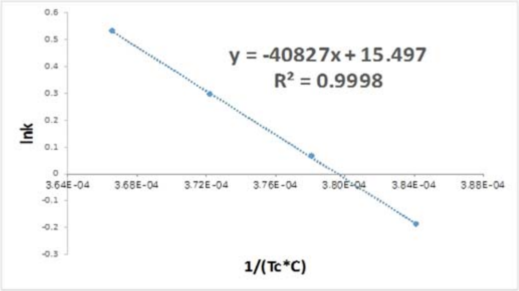

Supplementary Figure 29. Kinetic investigation of the hydrosilylation of n-hexene with triethoxysilane for activation energy. The experimental detail was provided in 2-5.

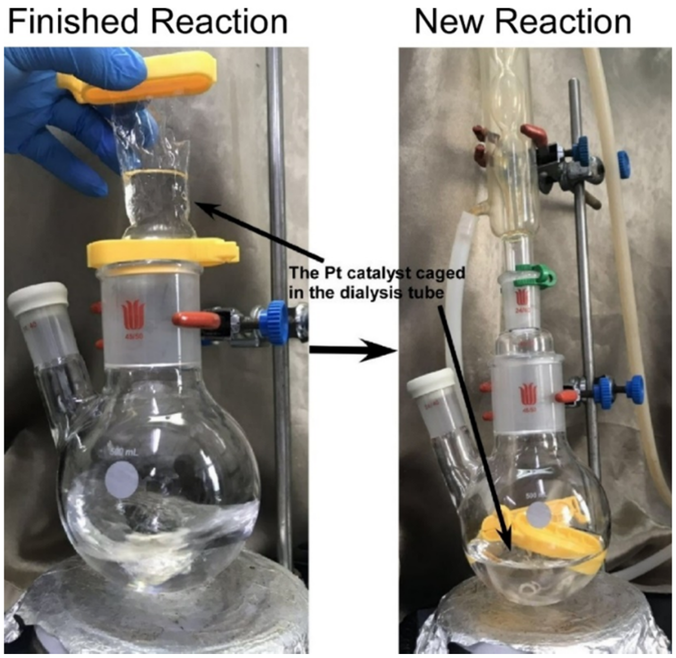

Supplementary Figure 30. Illustration of the **COP1-T-Pt** catalysts caged in dialysis tube (regenerated cellulose) with the molecular weight cutoff of 2k. The solution in the dialysis tube was the same as that in the flask, but with the presence of **COP1-T-Pt** catalyst. The reaction was conducted at 50 ^{o}C. Control experiments with Karstedt's catalyst within the dialysis tube resulted in complete activity loss during the second use.

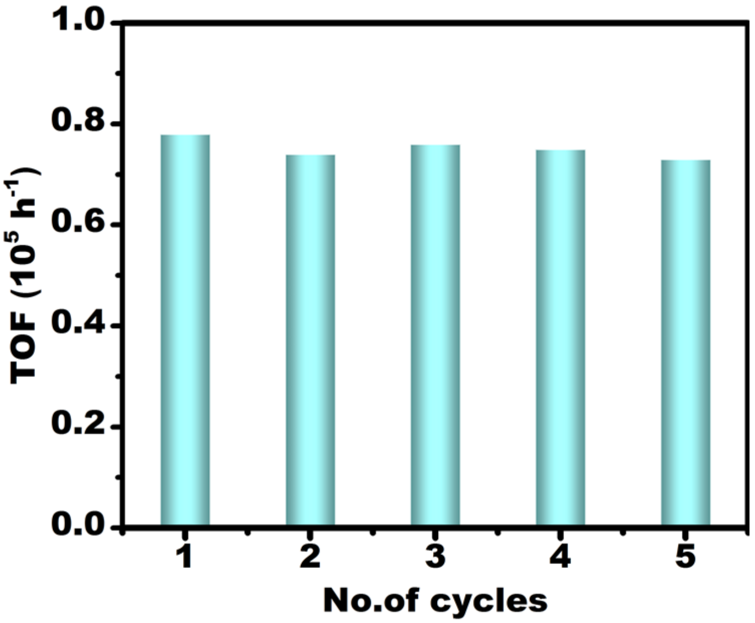

Supplementary Figure 31. Recyclability of the **COP1-T-Pt** catalyst caged in dialysis tube as supplementary Fig. 30 with the molecular weight cutoff of 2k.

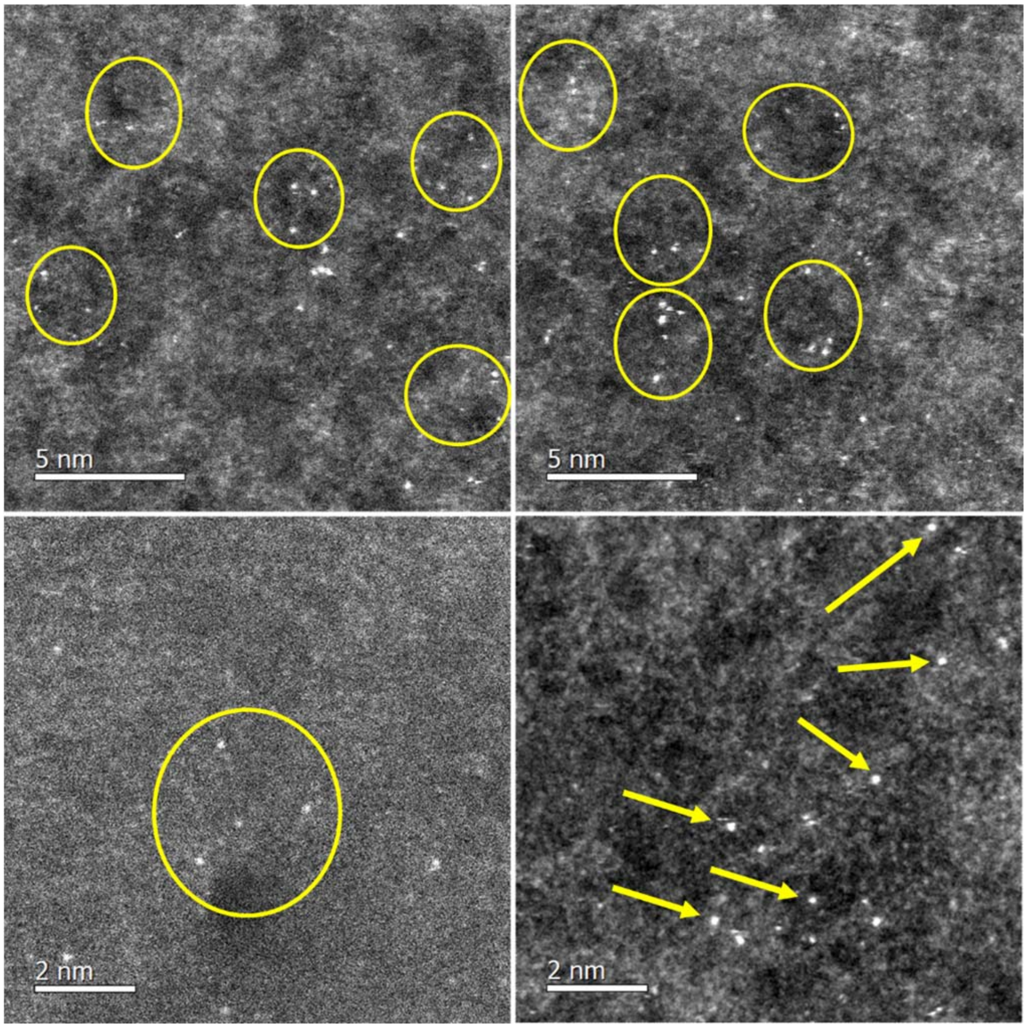

Supplementary Figure 32. ACHAADF-STEM image of **COP1-T-Pt** after five uses revealing the presence of single Pt atoms.

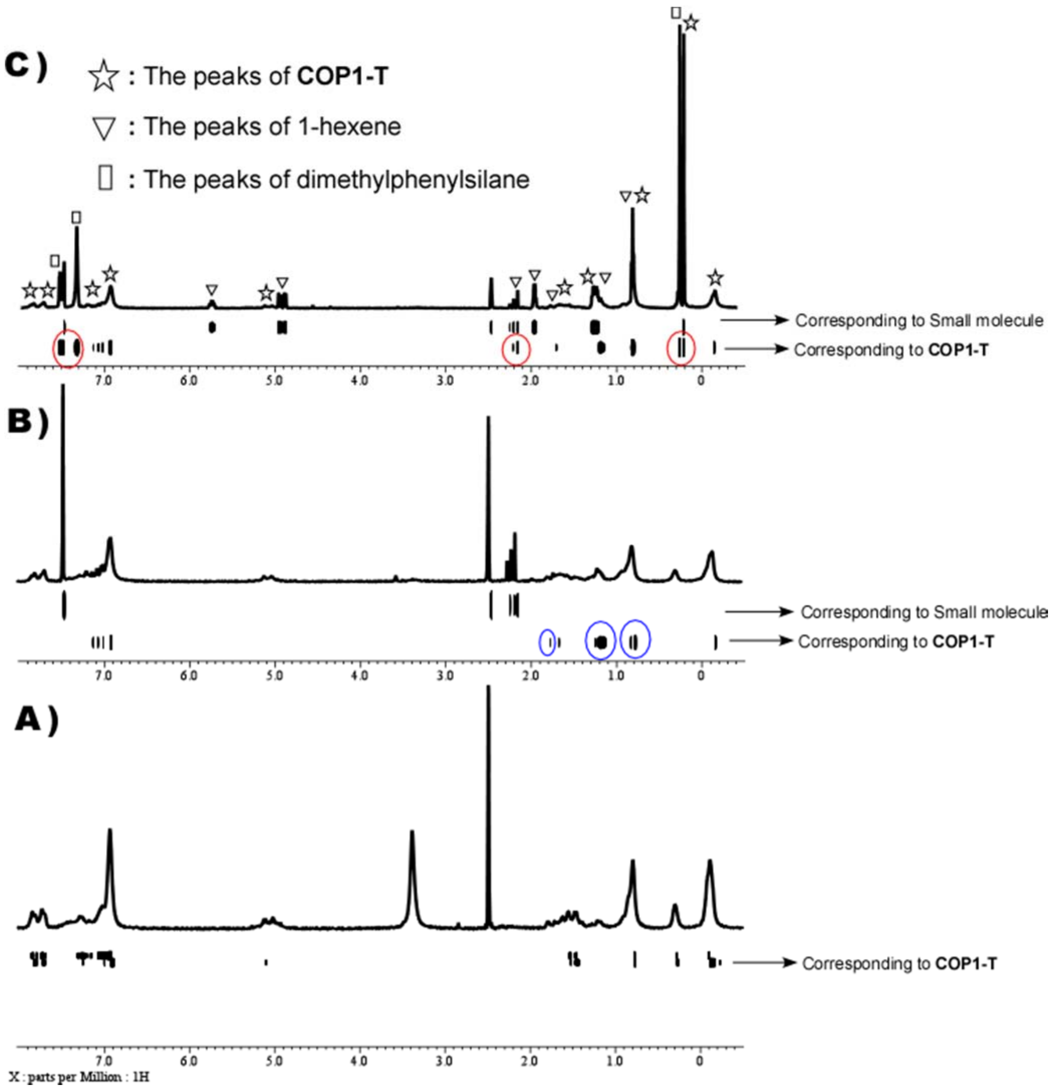

Supplementary Figure 33. ^{1}H-DOSY spectrum (600 MHz, DMSO-d6, 301 K) of **COP1-T** (A), **COP1-T** with 1-hexene (B) and **COP1-T** with 1-hexene and dimethylphenylsilane (C). The 1-hexene signals within **COP1-T** were highlighted with blue circles, and the dimethylphenylsilane signals within **COP1-T** were highlighted with red circles.

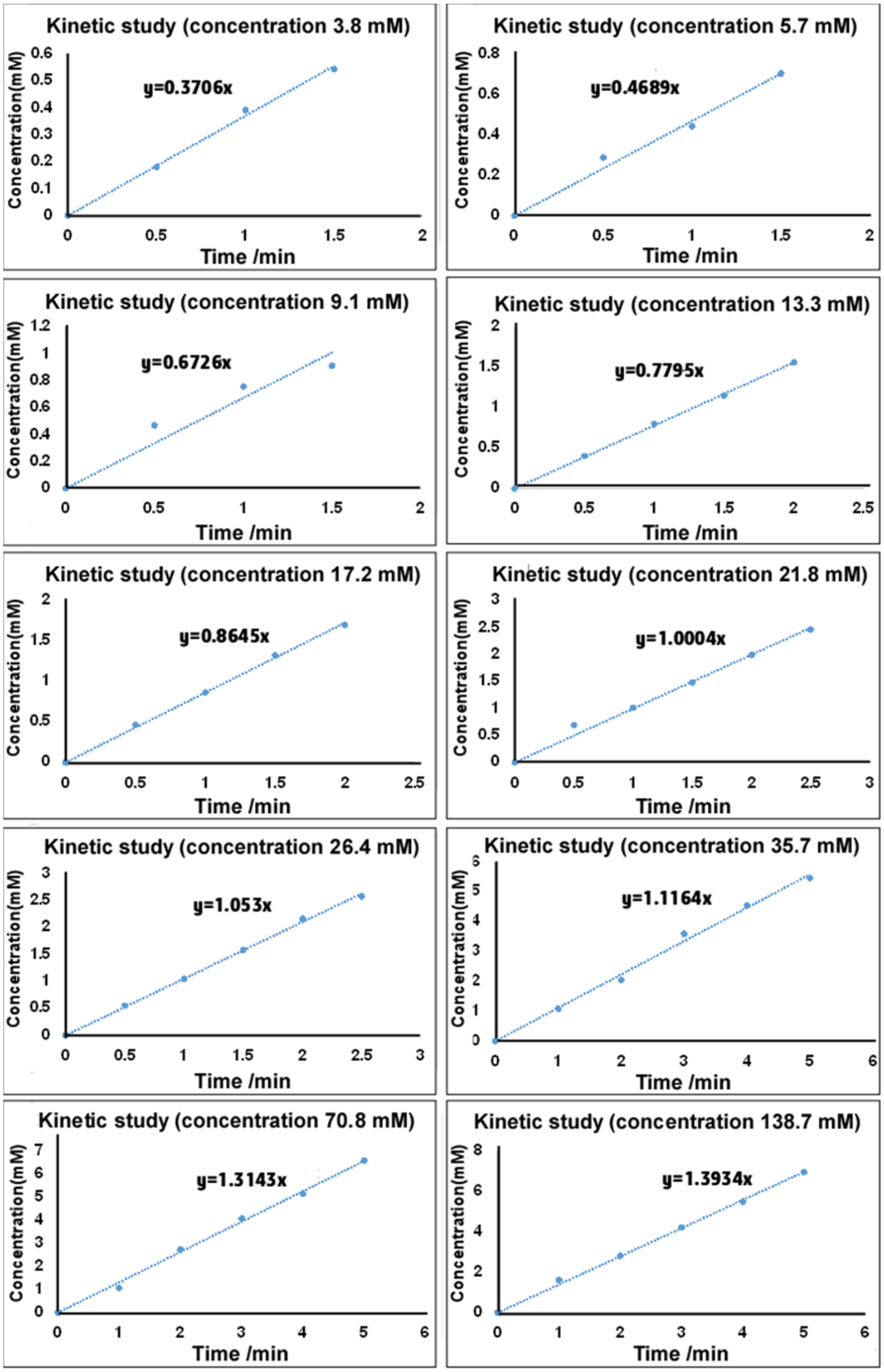

Supplementary Figure 34. The initial reaction rates of the hydrosilylation reaction of 1-hexene with different concentrations of triethoxysilane (3.7 mM, 5.6 mM, 9.1 mM, 13.3 mM, 17.2 mM, 21.8 mM, 26.4 mM, 35.7 mM, 70.8 mM, and 138.7 mM respectively) at room temperature under argon. The experimental details were provided in section 2-1.

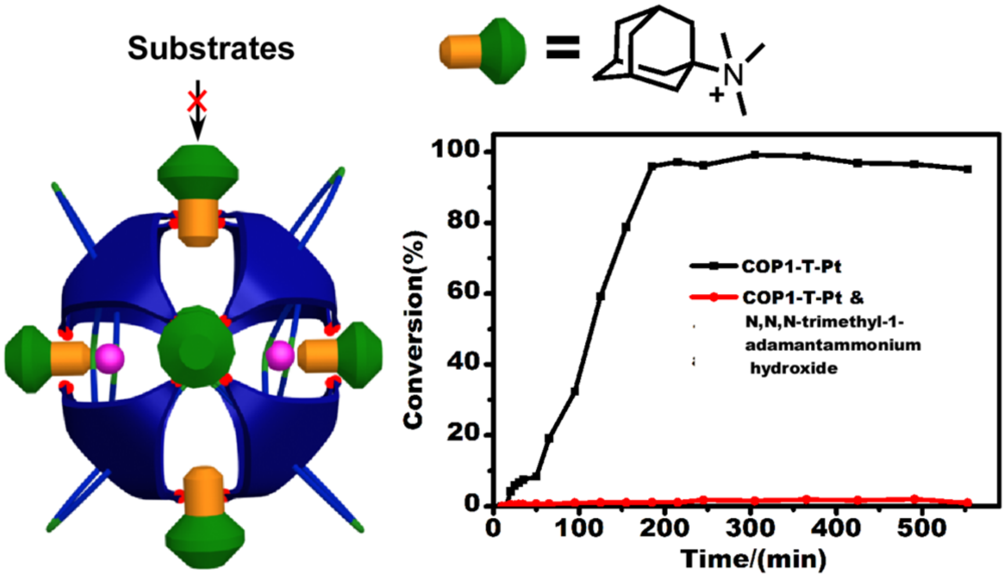

Supplementary Figure 35. The kinetic study for reactions with and without cage blocking. The cartoon picture (left) illustrates cage structure with the presence of bulky N,N,N-trimethyl-1-adamantammonium ions around. The reaction kinetic profile (right) shows that introducing bulky N,N,N-trimethyl-1-adamantammonium ions significantly reduces the reaction rate. The experimental detail for both reactions was provided in **2-2**.

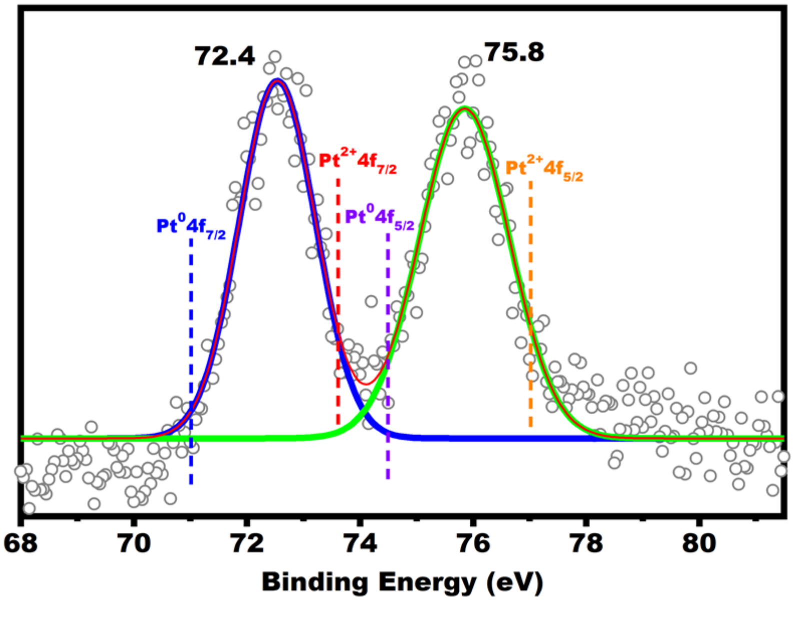

Supplementary Figure 36. The oxidation state of Pt in **COP1-T-Pt** was measured by X-ray photoelectron spectroscopy (XPS). The Pt 4f spectrum was deconvoluted into two peaks at binding energies of 75.8 and 72.4 eV, corresponding to $4f_{5/2}$ and $4f_{7/2}$ level, respectively. The peak positions are between those of Pt(II) and Pt(0), indicating Pt atoms carry partially positive charge(*J. Am. Chem. Soc.* **140**, 7407−7410 (2018))[11].

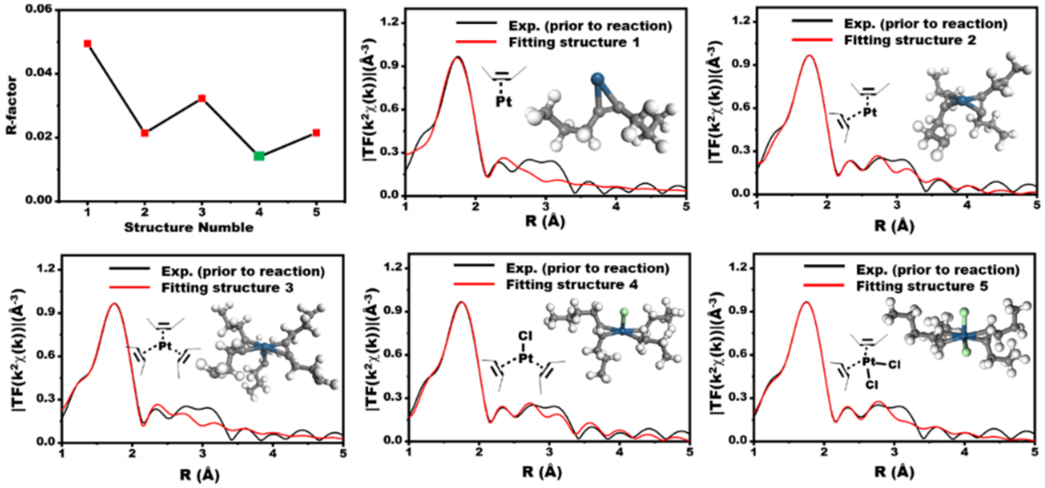

Supplementary Figure 37. The R-factors plot and the corresponding structural models for the **COP1-T-Pt** catalyst prior to reaction. The green dot represented the best model.

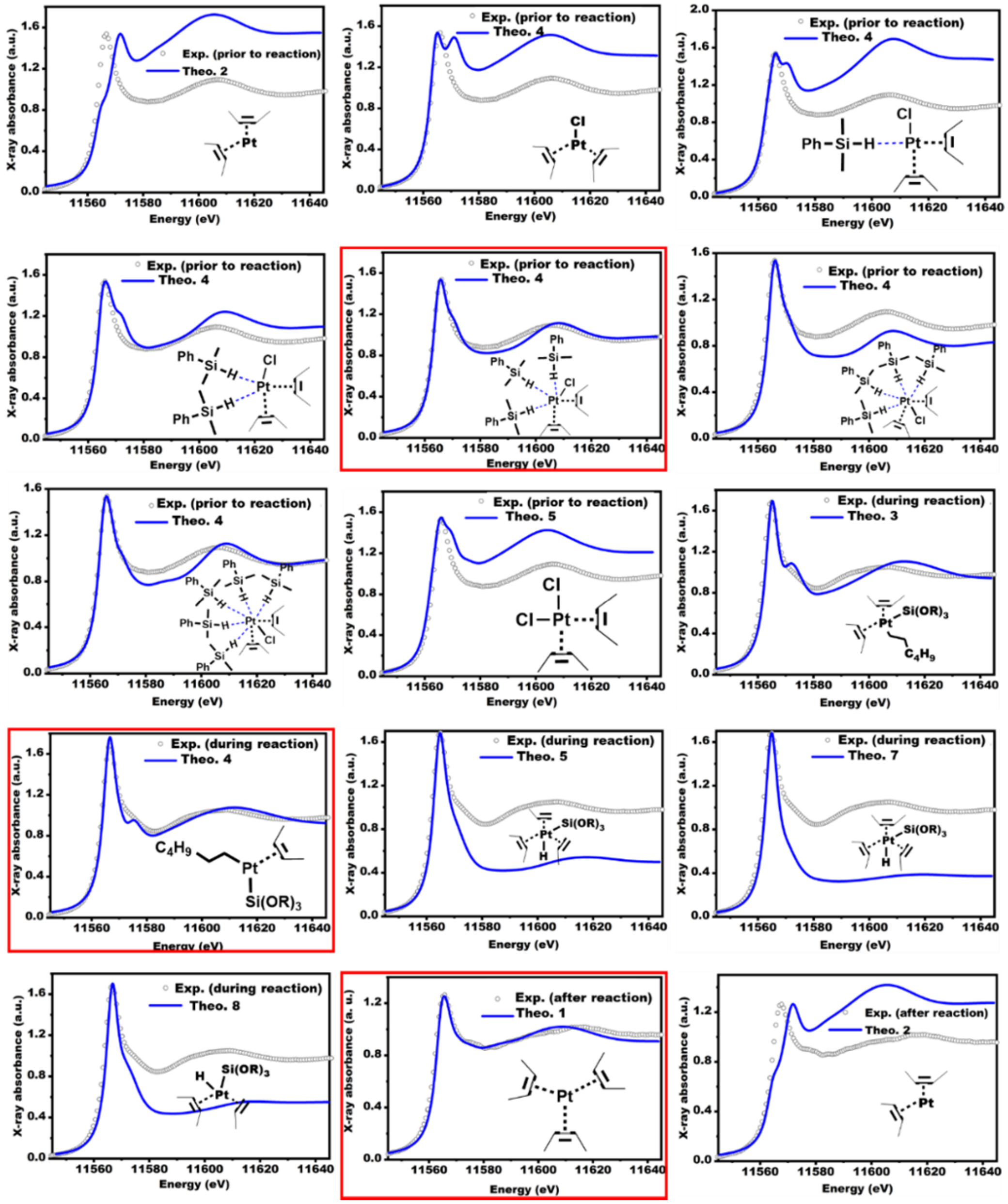

Supplementary Figure 38. The theoretical spectra calculated for the corresponding depicted structures which showed relatively small fitting R-factors during EXAFS fitting but not used in the manuscript. These simulated spectra for these models match less satisfactorily with the best models shown in Figure. 2h-2j. The one highlighted in red boxes are the best one.

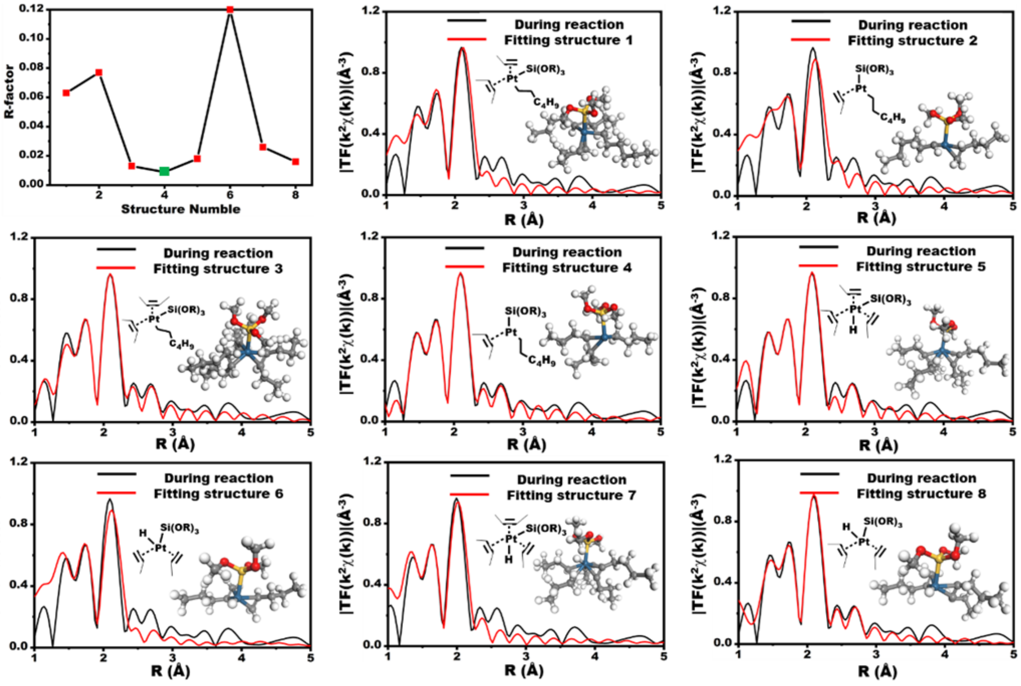

Supplementary Figure 39. The R-factors plot and the corresponding structural models for the **COP1-T-Pt** catalyst during reaction. The green dot represented the best model.

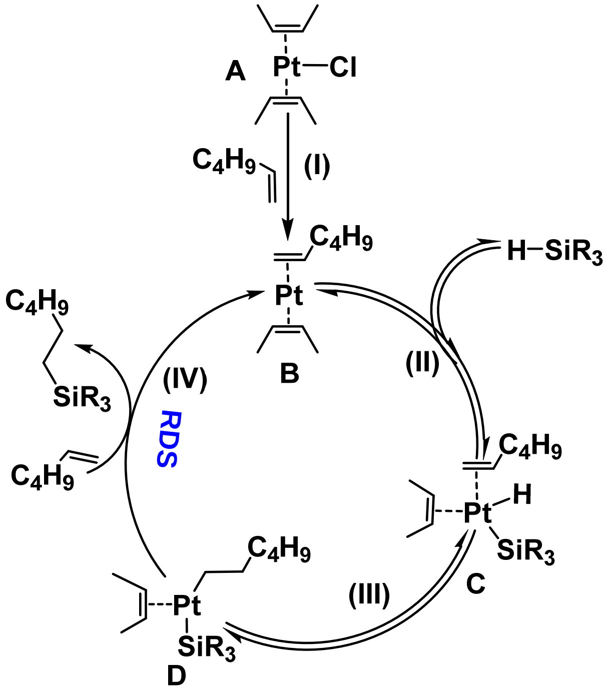

Supplementary Figure 40. The *Chalk-Harrod* reaction mechanism include four steps: (I) Transformation of A into zero-valence Pt species ; (II) Oxidative addition of $HSiR_3$; (III) Insertion of the olefin into the Pt–H bond; (IV) Reductive elimination of

alkylsilane. It is believed that step II and III are reversible and that step IV is the rate determining step (RDS).

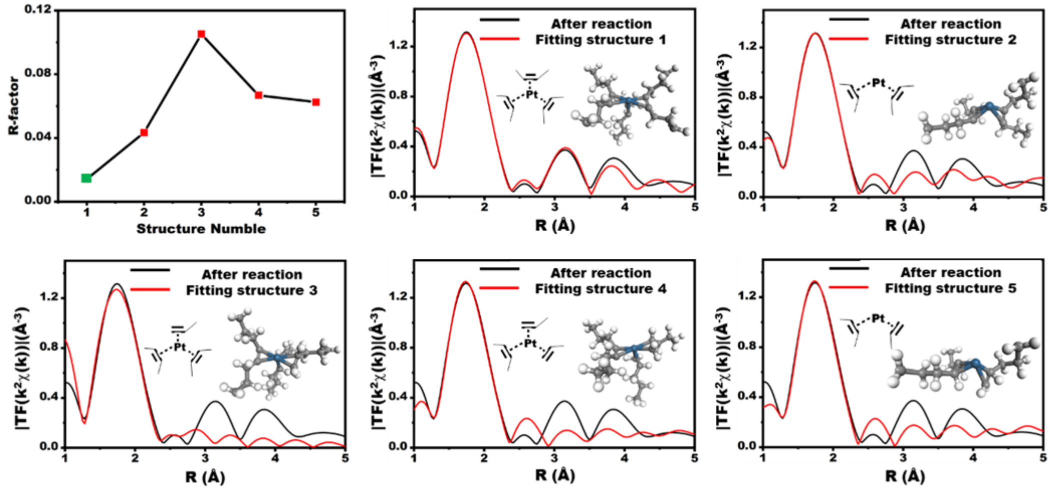

Supplementary Figure 41. The R-factor plot of the corresponding fitted structural models for the **COP1-T-Pt** catalyst after reaction. The green dot represented the selected the structure model.

Supplementary Table 2. Pt L3-edge EXAFS curve fitting parameters of **COP1-T-Pt** prior to reaction[a]

Structure NO.	path	N	R (Å)	σ^2 ($Å^2$)	ΔE0 (eV)	R
**1**	Pt-C1	1	2.0618	0.02809	3.263	0.049
	Pt-C2	1	2.1689	0.01178	15.796	
**2**	Pt-C1	1	2.01452	0.00806	-1.999	0.021
	Pt-C2	1	2.18480	0.01301	-0.500	
	Pt-C3	1	2.34217	0.00987	14.278	
	Pt-C4	1	2.96728	0.00778	18.779	
**3**	Pt-C1	1	1.9018	0.00547	-27.993	0.032
	Pt-C2	2	2.17408	0.00805	15.785	
	Pt-C3	2	2.16708	0.00191	0.994	
	Pt-C4	1	2.69111	0.0433	33.149	
**4**	Pt-C1	1	2.1277	0.01208	1.843	**0.014**
	Pt-C2	2	2.18428	0.00917	1.554	
	Pt-C3	1	2.52692	0.00914	8.811	
	Pt-Cl	1	2.67423	0.00097	-9.806	
**5**	Pt-C1	2	2.23997	0.0006	28.805	0.022
	Pt-C2	2	2.5028	0.01466	6.164	
	Pt-Cl	2	2.4998	0.01038	3.582	

Supplementary Table 3. Pt L3-edge EXAFS curve fitting parameters of **COP1-T-Pt** during reaction[b]

Structure NO.	path	N	R (Å)	σ^2 ($Å^2$)	ΔE0 (eV)	R
**1**	Pt-C	2	2.0859	0.00407	13.957	0.063
	Pt-C	1	2.1481			
	Pt-C	1	2.4065			
	Pt-C	1	2.4066			
	Pt-Si	1	2.4215	-0.00106	-8.048	
**2**	Pt-C	1	1.9823	0.04576	4.333	0.077
	Pt-C	2	2.1351			
	Pt-Si	1	2.4374	-0.0027	-9.340	
**3**	Pt-C	2	2.0172	0.00450	7.048	0.013
	Pt-C	2	2.1946			
	Pt-C	1	2.3343			
	Pt-Si	1	2.6984	0.01164	21.024	
**4**	Pt-C	1	2.0034	0.00444	9.438	**0.0089**
	Pt-C	1	2.1578			
	Pt-C	1	2.3201			
	Pt-Si	1	2.6353	0.00732	13.890	
**5**	Pt-H	1	1.7899	-0.01255	28.809	0.018
	Pt-C	2	2.0916	-0.00121	12.635	
	Pt-C	1	2.2058			
	Pt-C	2	2.4095			
	Pt-Si	1	2.3877	-0.00445	-13.942	
**6**	Pt-H	1	1.7164	0.08675	137.160	0.12
	Pt-C	1	2.1002	0.11459	30.247	
	Pt-C	2	2.1463			
	Pt-C	1	2.1741			
	Pt-Si	1	2.5129	-0.00042	5.052	
**7**	Pt-H	1	1.5854	-0.01054	-154.852	0.026
	Pt-C	2	1.9665	-0.00572	12.049	
	Pt-C	1	2.1488			
	Pt-C	2	2.3295			
	Pt-Si	1	2.5123	-0.00193	-12.858	
**8**	Pt-H	1	1.80445	-0.00900	28.808	0.016
	Pt-C	1	2.06050	0.00499	11.454	
	Pt-C	2	2.14701			
	Pt-C	1	2.33016			
	Pt-Si	1	2.54488	0.00704	3.662	

Supplementary Table 4. Pt L3-edge EXAFS curve fitting parameters of **COP1-T-Pt** after reaction[c]

Structure NO.	path	N	R (Å)	σ^2 (Å^2)	ΔE0 (eV)	R
**1**	Pt-C1	1.2	1.78287	0.02849	-7.649	**0.014**
	Pt-C2	1.2	1.96128	0.02935	10.275	
	Pt-C3	2.4	2.06692	0.02434	-11.873	
	Pt-C4	1.1	3.10109	0.01602	5.0762	
**2**	Pt-C1	1	1.68207	-0.03886	7.086	0.043
	Pt-C2	1	2.07118	0.04216	-10.802	
	Pt-C3	1	1.97939	0.04261	11.881	
	Pt-C4	1	2.37918	-0.04041	-6.1023	
**3**	Pt-C1	2	2.0194	0.01709	16.225	0.11
	Pt-C2	2	2.10342	0.00144	-20.641	
	Pt-C3	2	2.11366	-0.01002	-14.081	
**4**	Pt-C1	2	1.95432	-0.02569	15.175	0.067
	Pt-C2	2	2.06674	0.02729	-8.575	
	Pt-C3	2	2.18375	0.0286	2.781	
**5**	Pt-C1	1	1.97684	-0.02726	14.97	0.063
	Pt-C2	1	2.09108	-0.02966	0.48	
	Pt-C3	2	2.20656	-0.02938	-12.622	

N, coordination number; *R*, distance between absorber and backscatter atoms; σ^2, Debye-Waller factor to account for both thermal and structural disorders; ΔE_0, inner potential correction; *R* factor indicates the goodness of the fit. Error bounds (accuracies) that characterize the structural parameters obtained by EXAFS spectroscopy are estimated as $N \pm 20\%$; $R \pm 1\%$; $\sigma^2 \pm 20\%$; $\Delta E_0 \pm 20\%$. $S0_2$ was fixed to 1.0 as determined from Pt foil fitting. Coordination number (*N*) fixed according to the crystal structure. [a]Fitting range: $3.0 \leq k$ (/Å) ≤ 11.9 and $1.2 \leq R$ (Å) ≤ 3.5. [b]The path parameters ΔE_0 and σ^2 of the same atoms were set uniform to reduce the fitting parameters due to the multishell of Pt catalyst during reaction. Fitting range: $2 \leq k$ (/Å) ≤ 12.5 and $1.2 \leq R$ (Å) ≤ 4. [c]Fitting range: $3.0 \leq k$ (/Å) ≤ 10.2 and $1.2 \leq R$ (Å) ≤ 3.5. The R-factor should be stated that: R-factor<0.02 means the model is good, 0.02<R-factor<0.05 means the models slightly deviate or data quality is not good enough, 0.05<R-factor<0.1 means the models greatly deviate or data quality is bad, R-factor >0.1 means the models is wrong. Reduced chi-square can't represent the fitting quality, so it wasn't shown here. All the fitting variables using custom guess method.

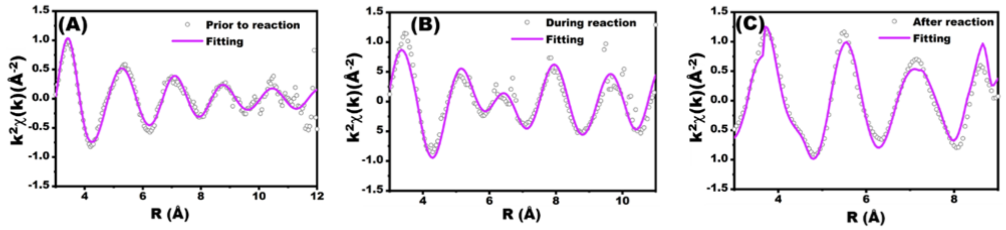

Supplementary Figure 42. A, B, C) The structures and quantitative EXAFS fitting curves at K space for **COP1-T-Pt** prior to reaction, during reaction and after reaction.

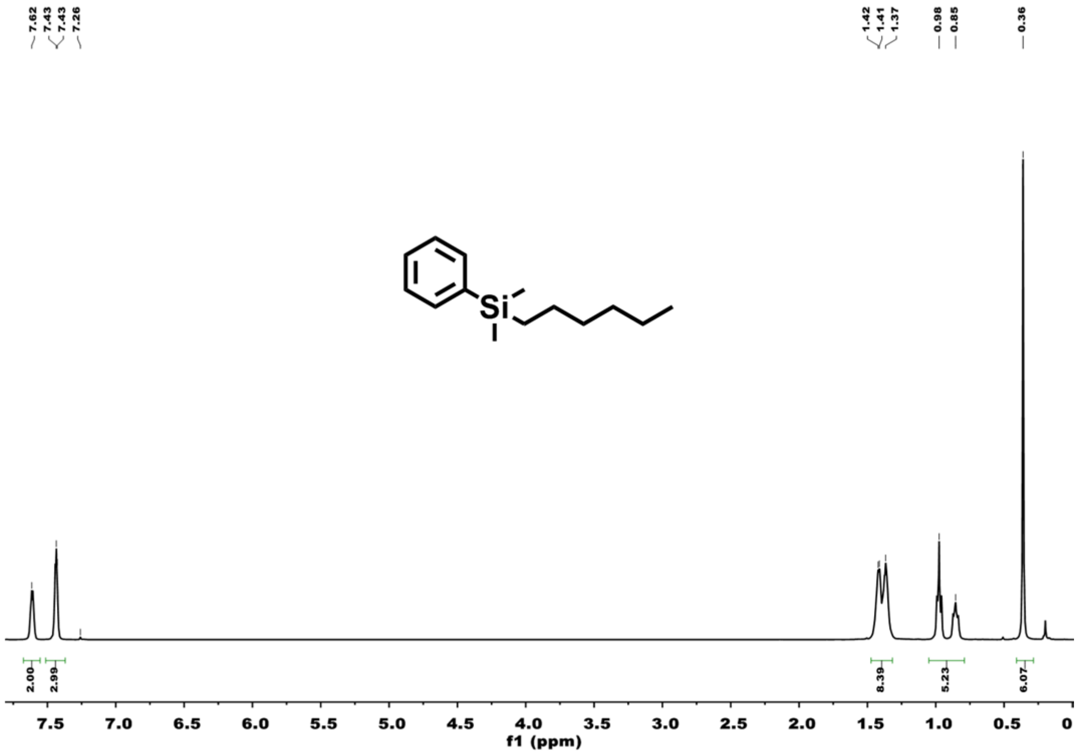

Supplementary Figure 43. ^{1}H-NMR of compound **2**

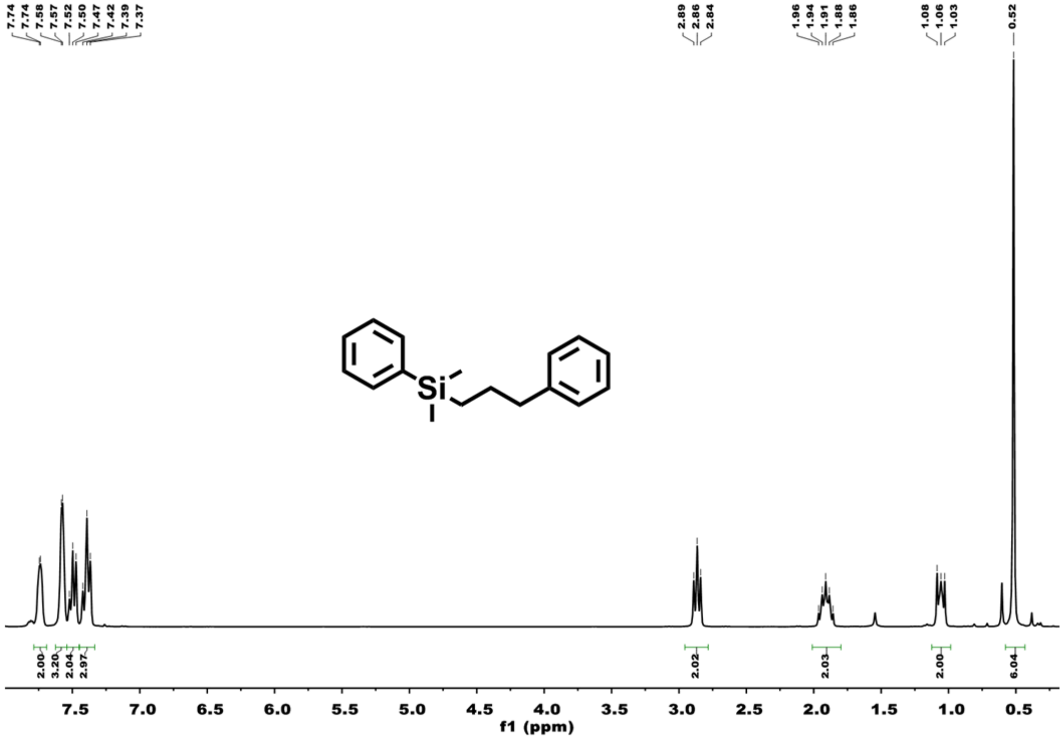

Supplementary Figure 44. ^{1}H-NMR of compound **3**

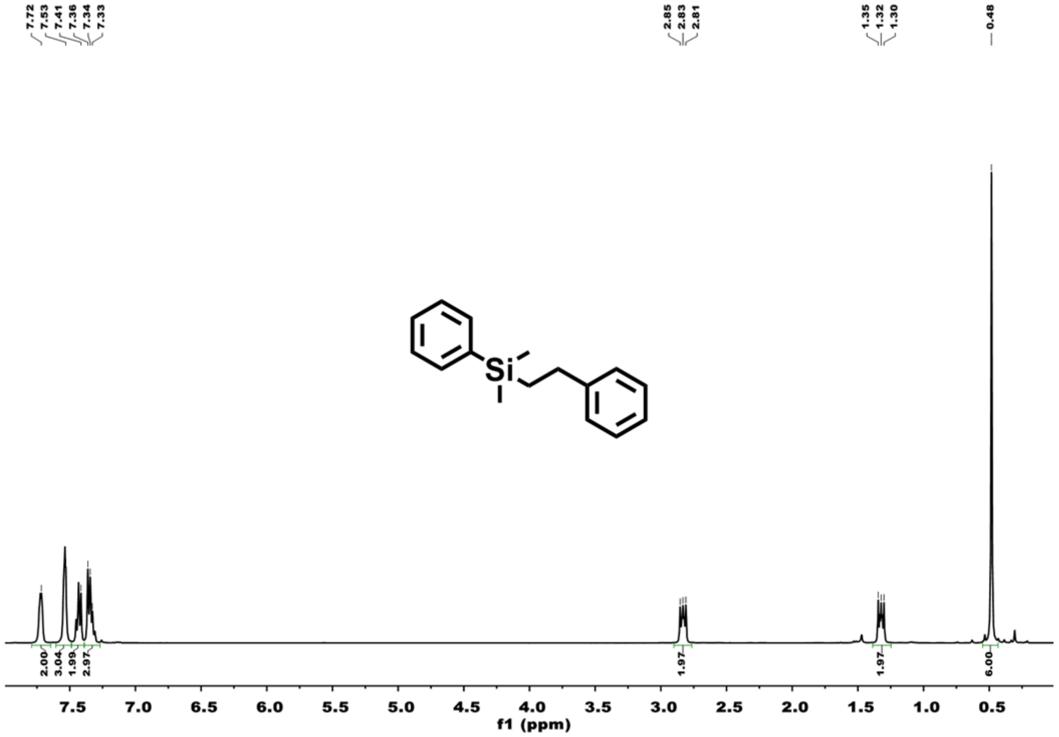

Supplementary Figure 45. ^{1}H-NMR of compound **4**

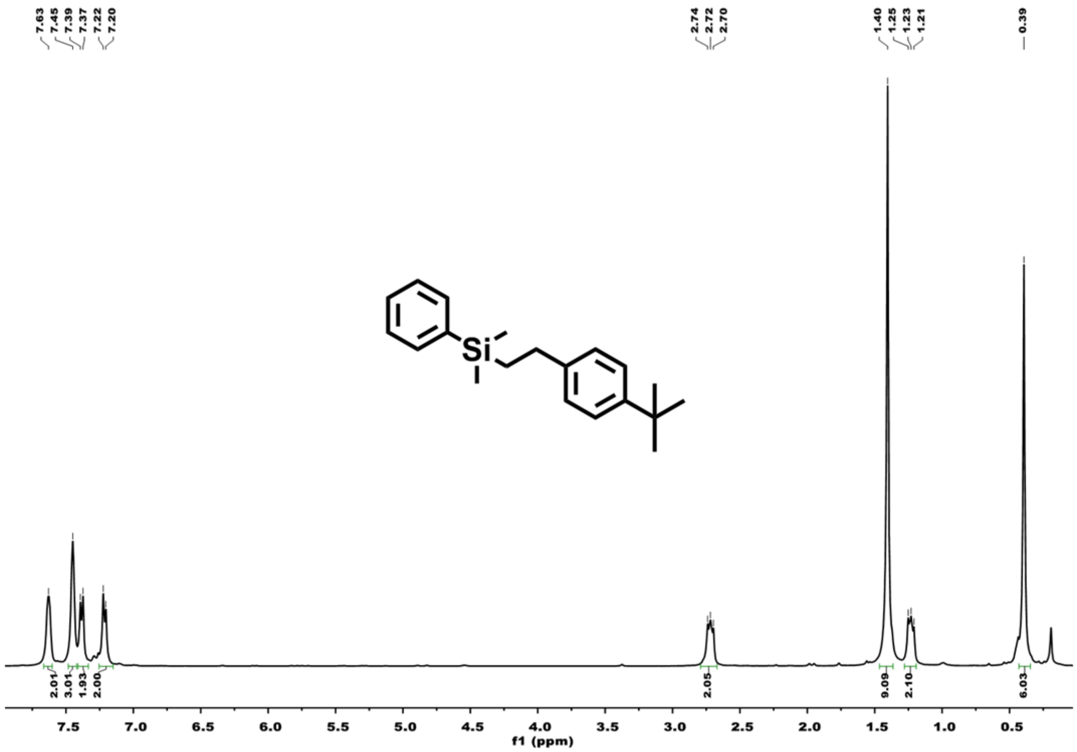

Supplementary Figure 46. ^{1}H-NMR of compound **5**

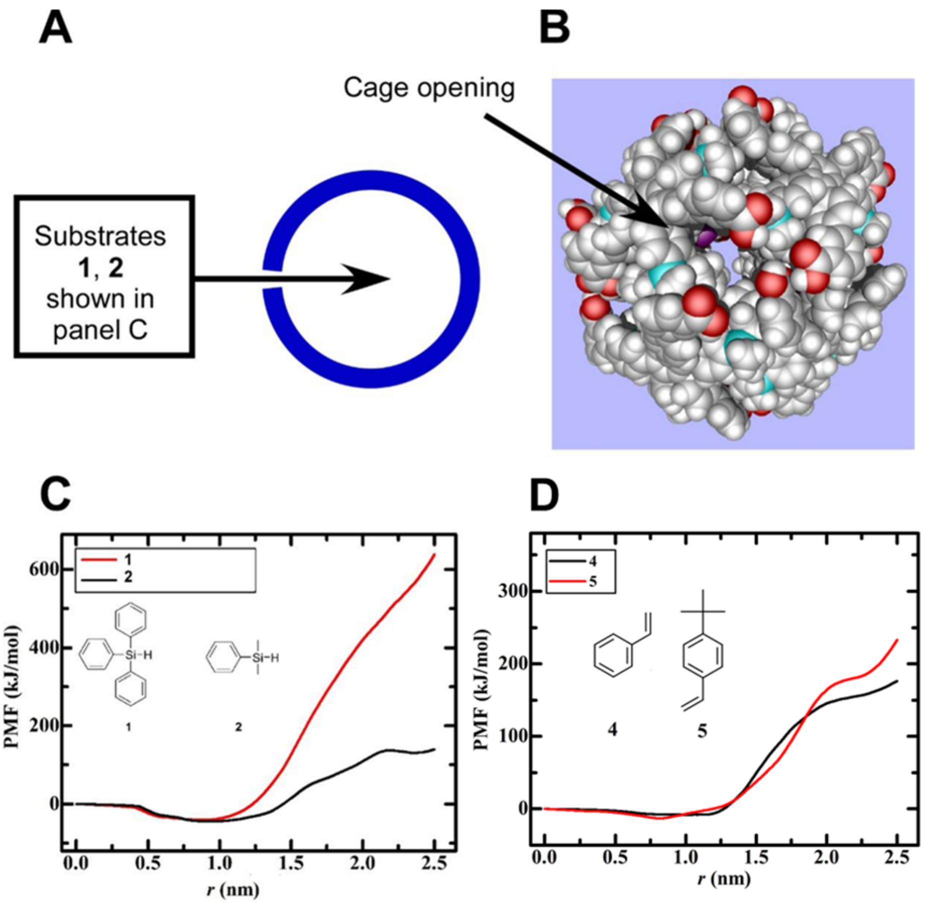

Supplementary Figure 47. Potential of mean force (PMF) calculated from pulling along the reaction coordinates (*r*) defined from outside to the center of the cage catalyst (A). Starting point of the reaction coordinate was located 2.5 nm away from the cage center. Initial structure model of the cage catalyst was built from the single-crystal structure of **MOP1** using the software of Diamond with the following procedure. First, the adjacent alkene bonds were metathesized, and then copper ions were removed to construct the model of **COP1-T**. Then four Pt atoms were inserted into the cage, and coordination between alkene and Pt atoms were established by making bonds between the alkene groups and Pt atoms. Three alkenes were made to connect to each Pt atom. The obtained structure was finally optimized to converge at PM7 level through the use of the software of MOPAC to get the final structure of **COP1-T-Pt** (B). (C) It showed that it took the larger triphenyl silane more energy to reach the center through the opening than dimethylphenyl silane. (D) Theoretical calculation showing the effect of the tertbutyl group when passing through the pore of **COP1-T-Pt** . It should be noted that the simulation can only qualitatively characterize the difficulty of molecules entering the cage catalyst.

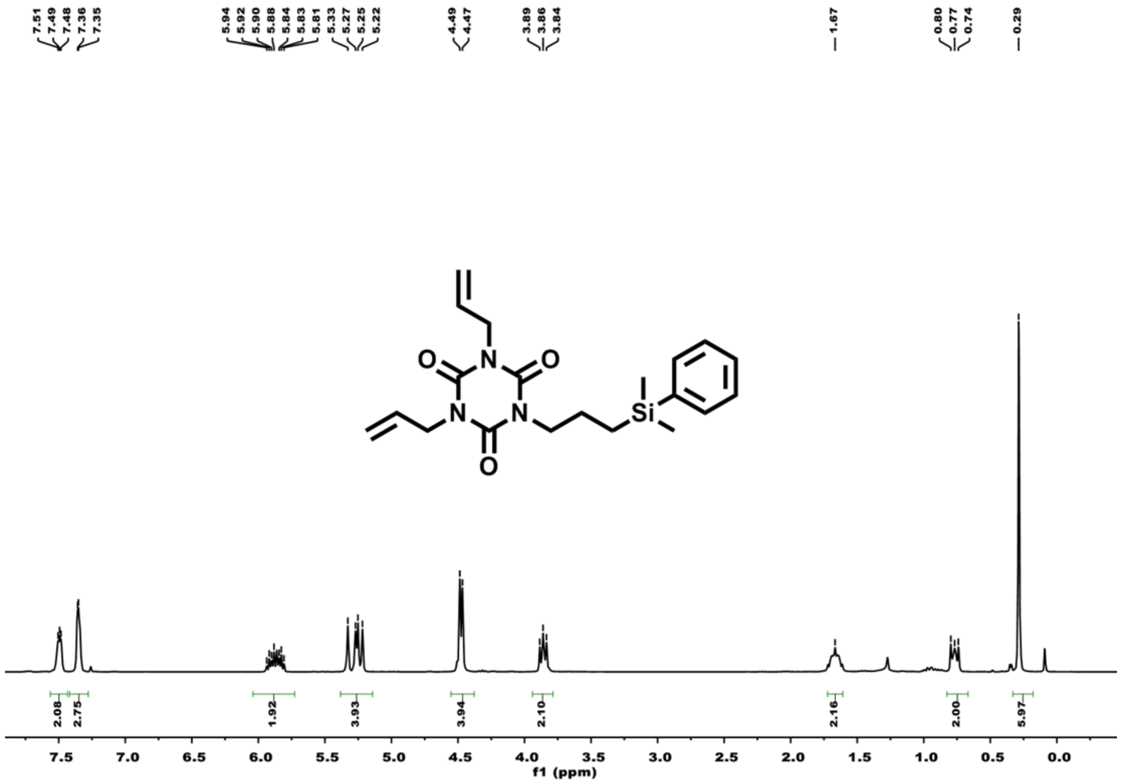

Supplementary Figure 48. ^{1}H-NMR of compound **12**

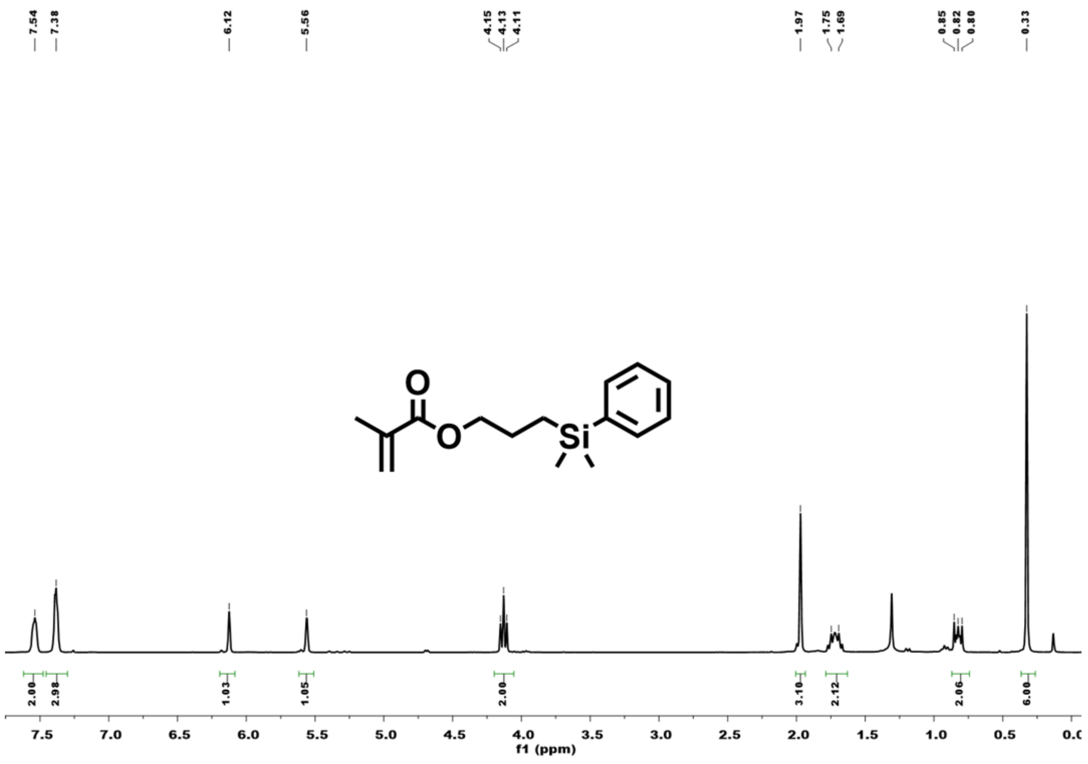

Supplementary Figure 49. ^{1}H-NMR of compound **13**

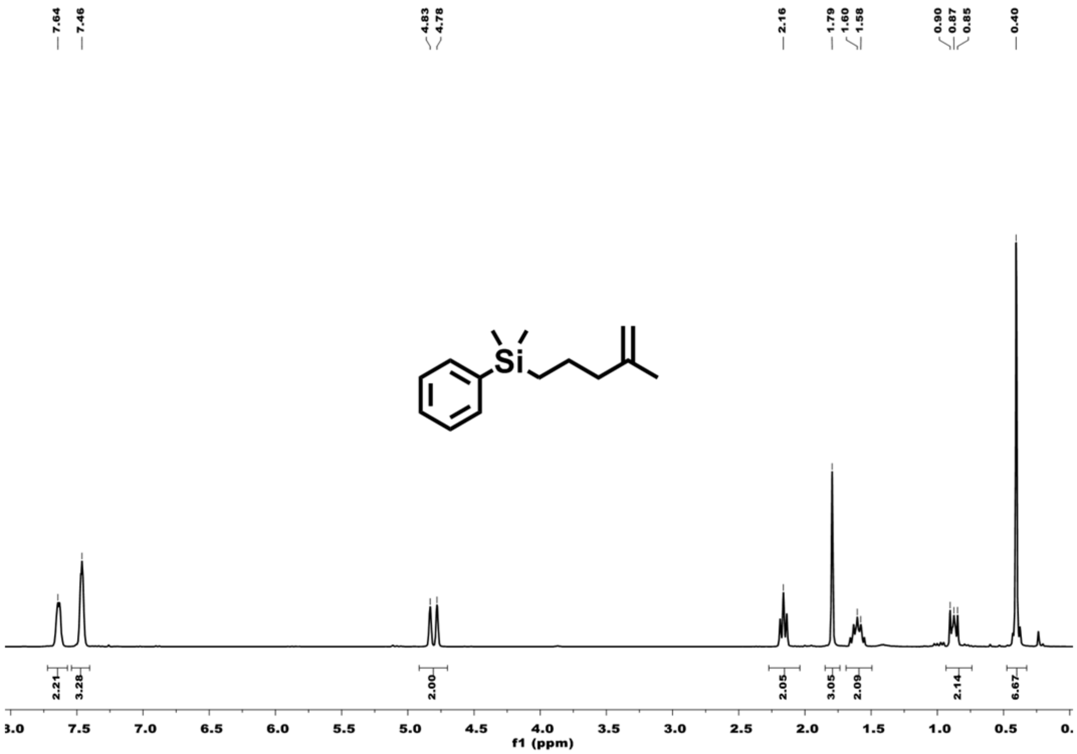

Supplementary Figure 50. ^{1}H-NMR of compound **14**

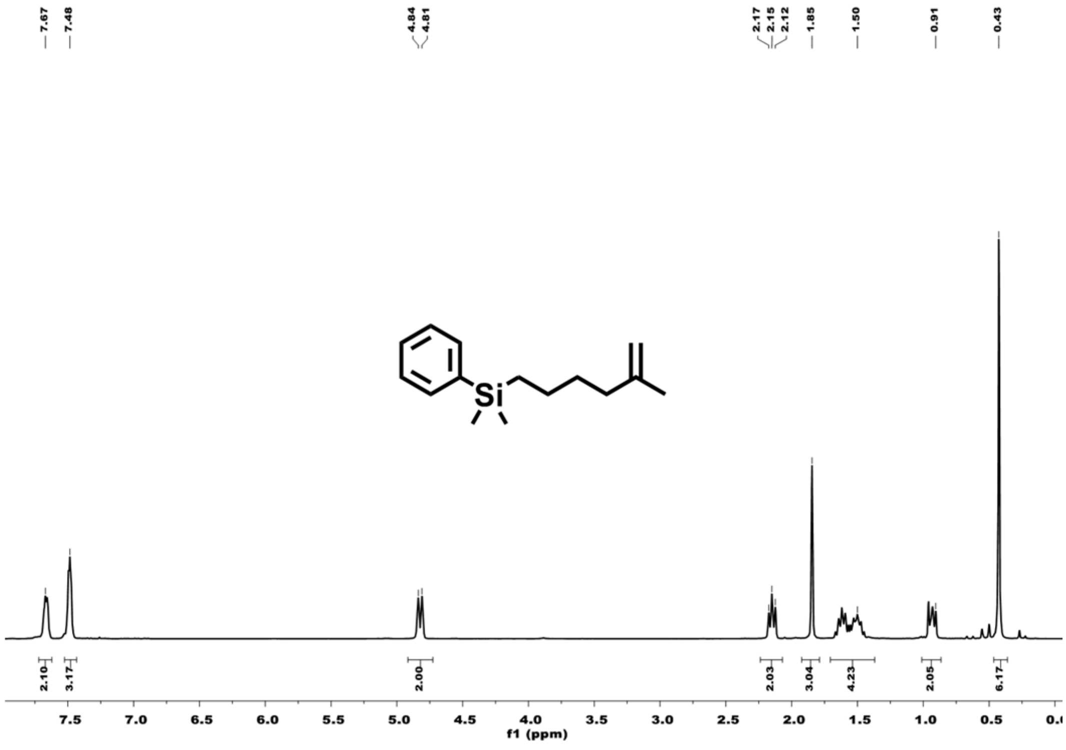

Supplementary Figure 51. ^{1}H-NMR of compound **15**

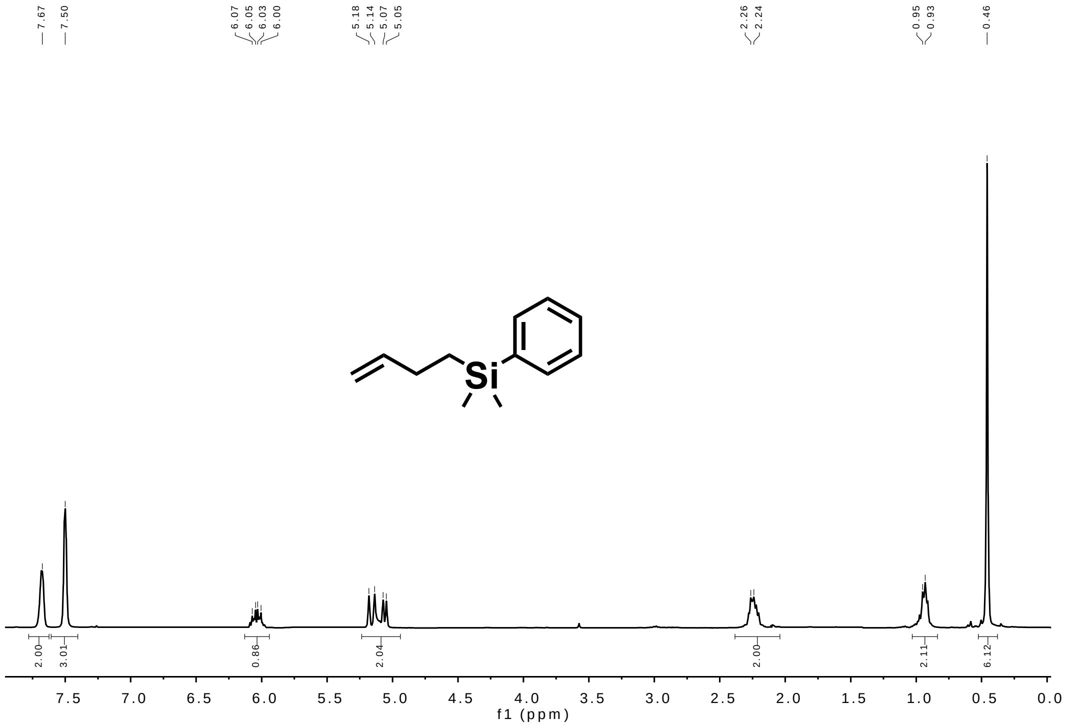

Supplementary Figure 52. ^{1}H-NMR of compound **16**

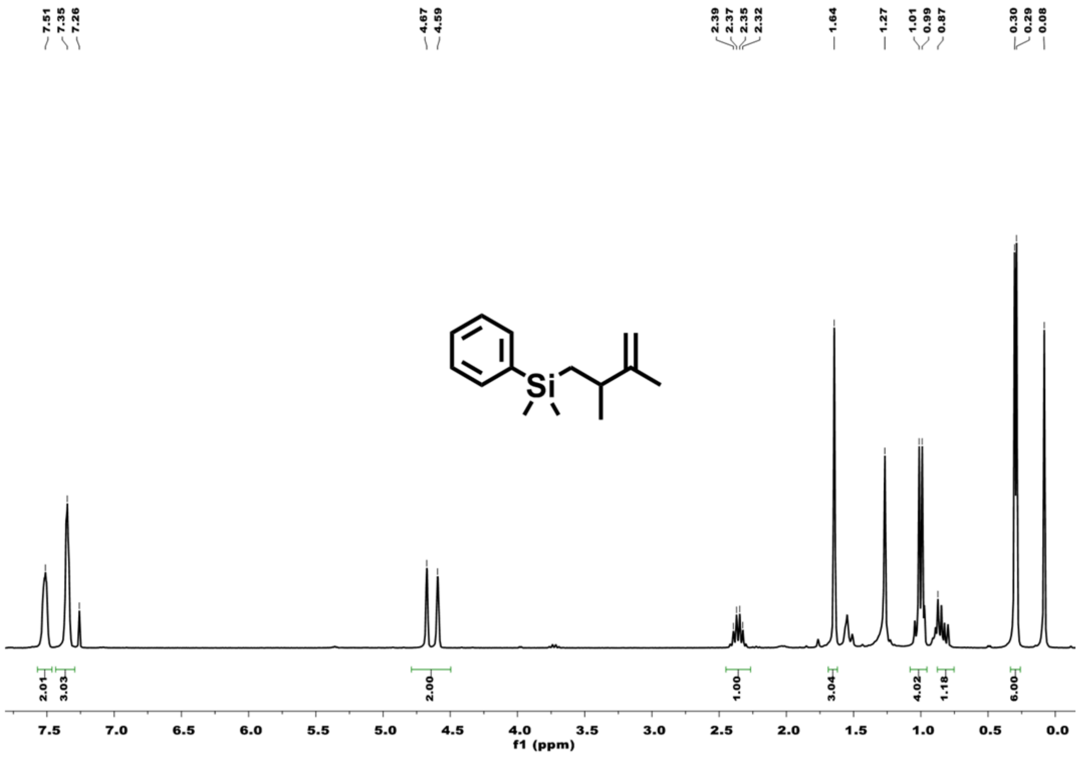

Supplementary Figure 53. ^{1}H-NMR of compound **17**

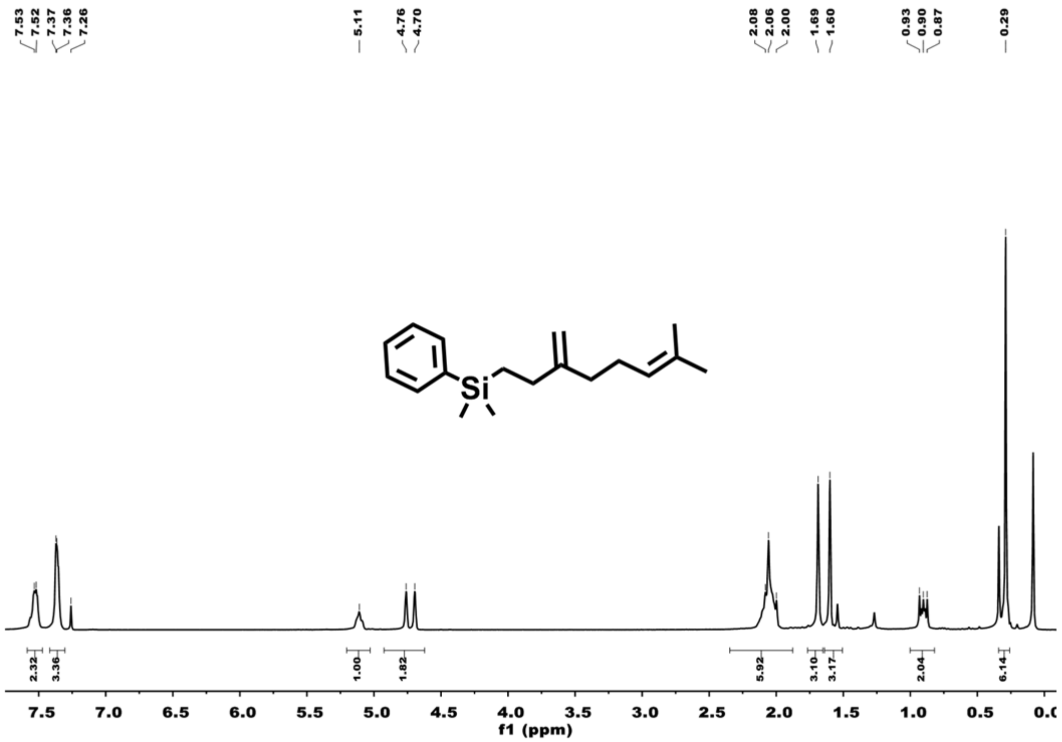

Supplementary Figure 54. ^{1}H-NMR of compound **18**

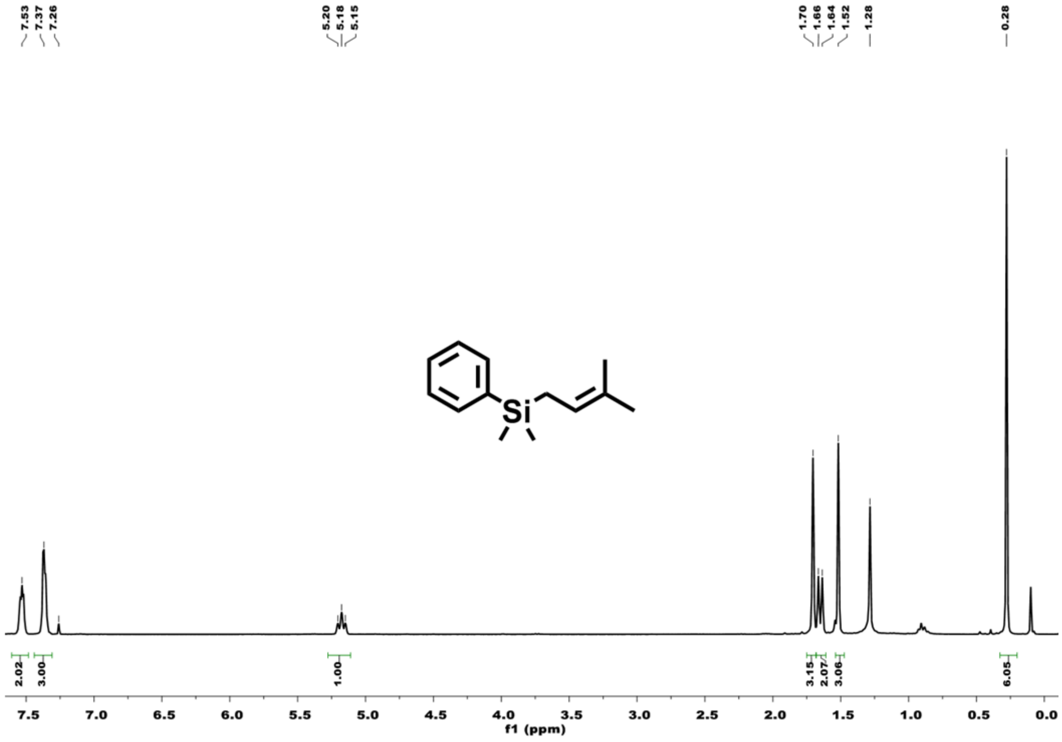

Supplementary Figure 55. ^{1}H-NMR of compound **19**

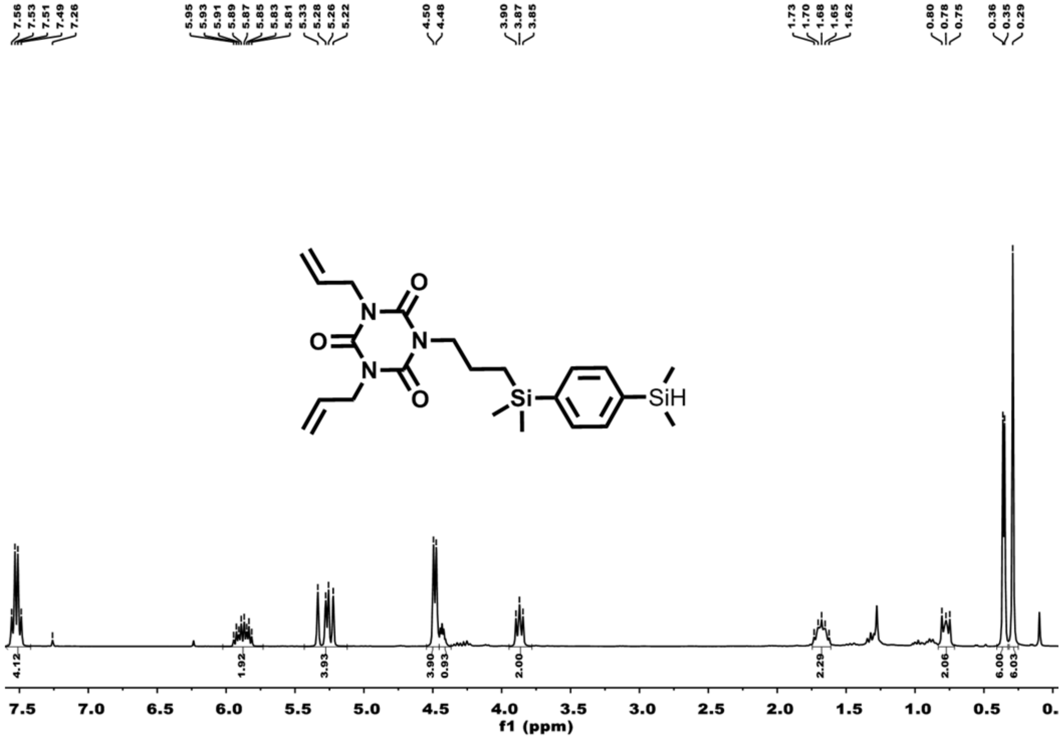

Supplementary Figure 56. ^{1}H-NMR of compound **20**

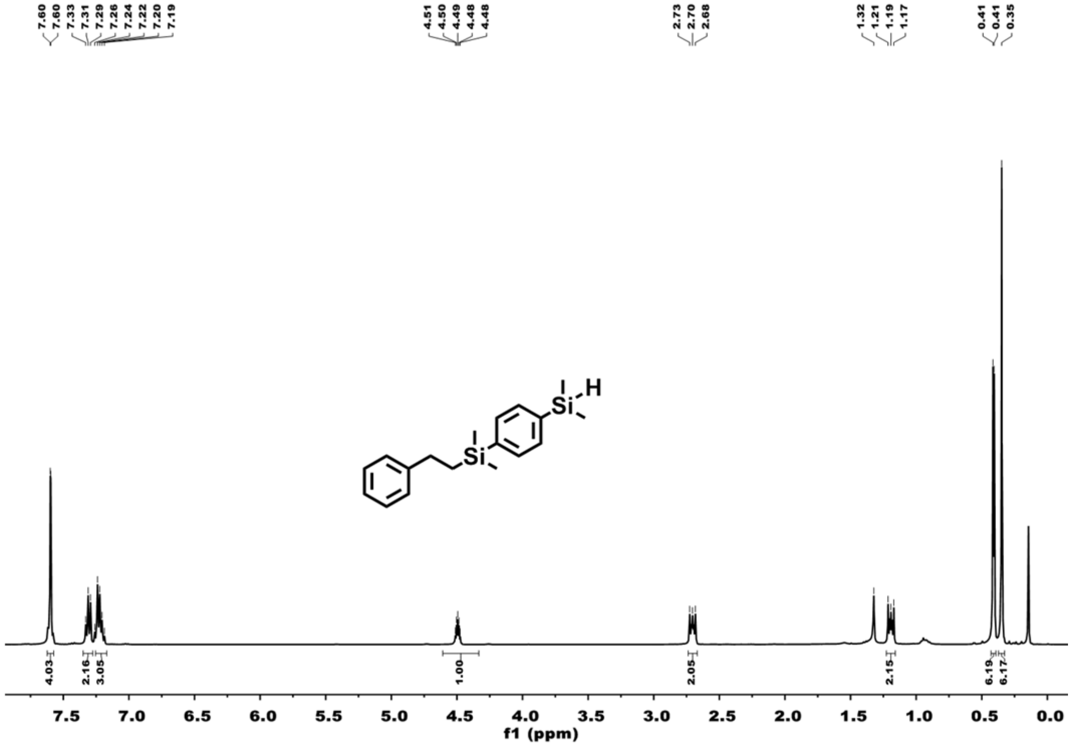

Supplementary Figure 57. ^{1}H-NMR of compound **21**

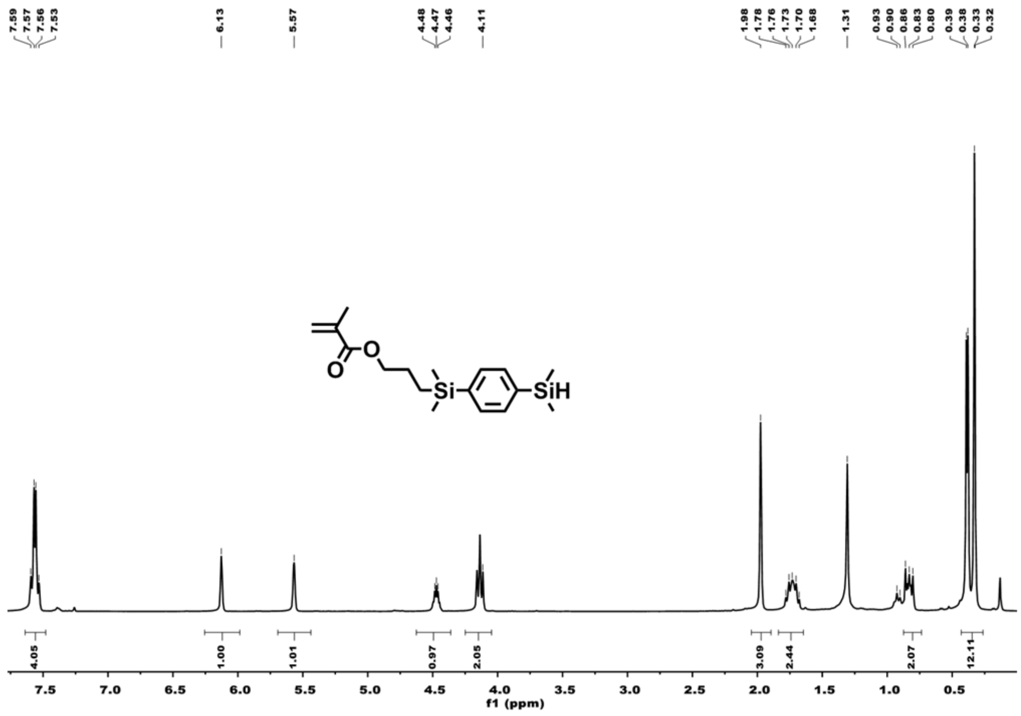

Supplementary Figure 58 ^{1}H-NMR of compound **22**

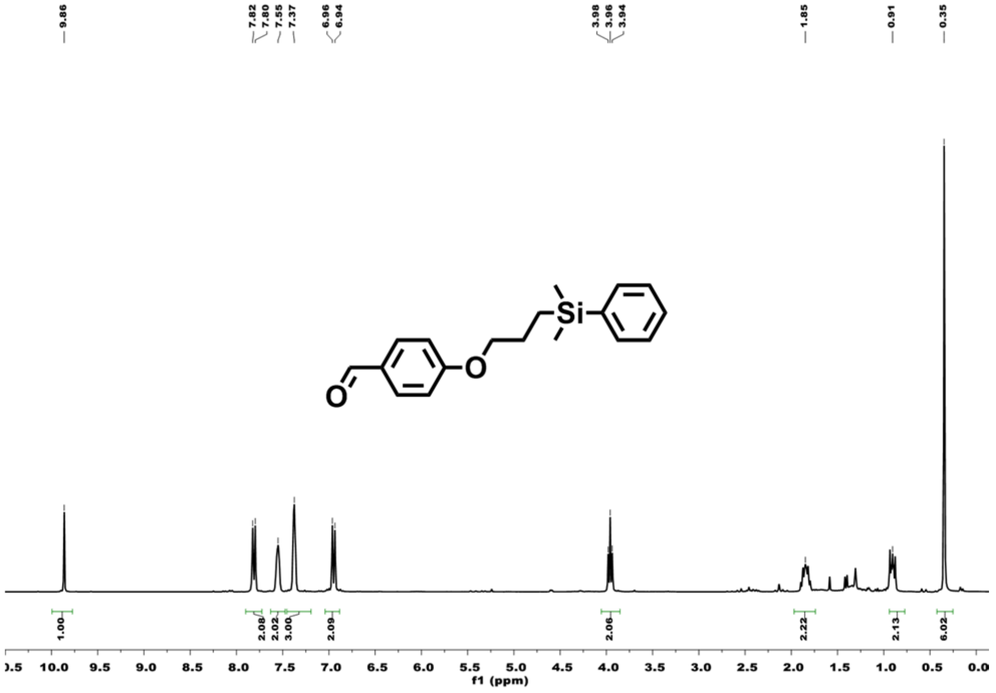

Supplementary Figure 59. ^{1}H-NMR of compound **23**

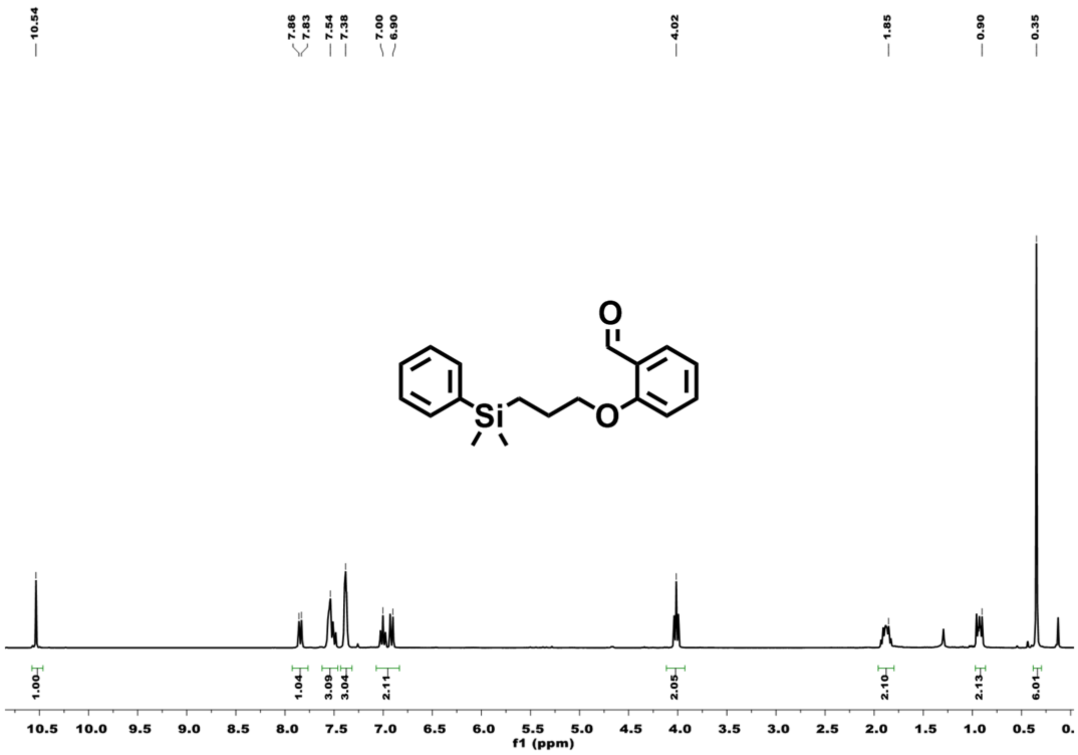

Supplementary Figure 60. ^{1}H-NMR of compound **24**

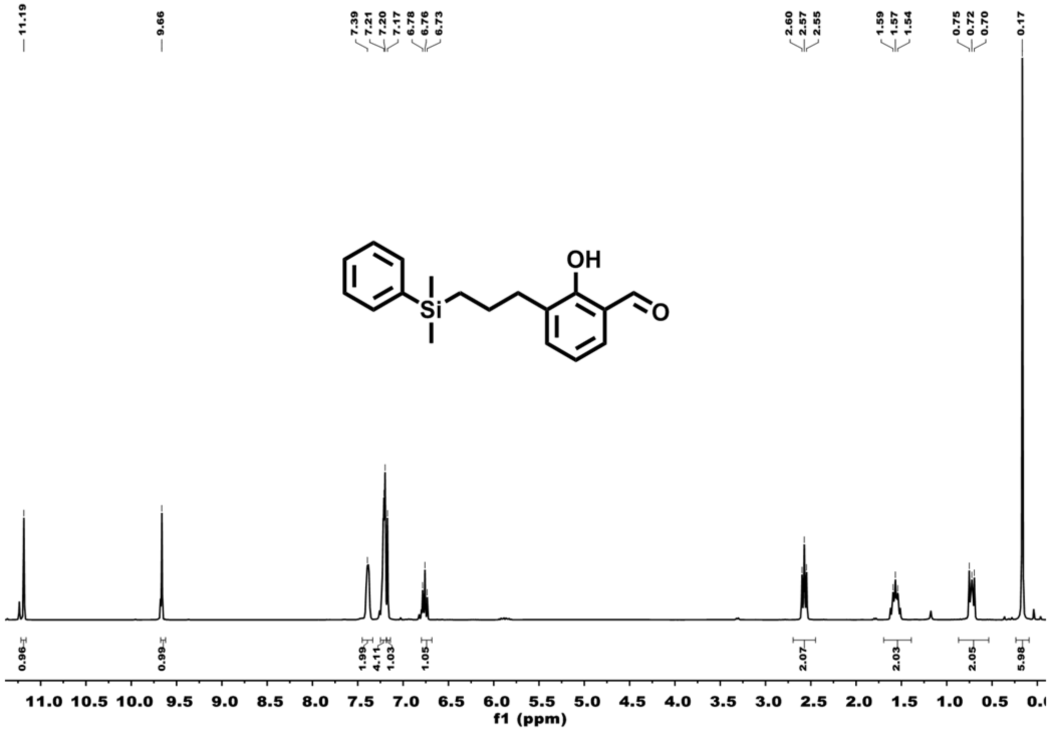

Supplementary Figure 61. ^{1}H-NMR of compound **25**

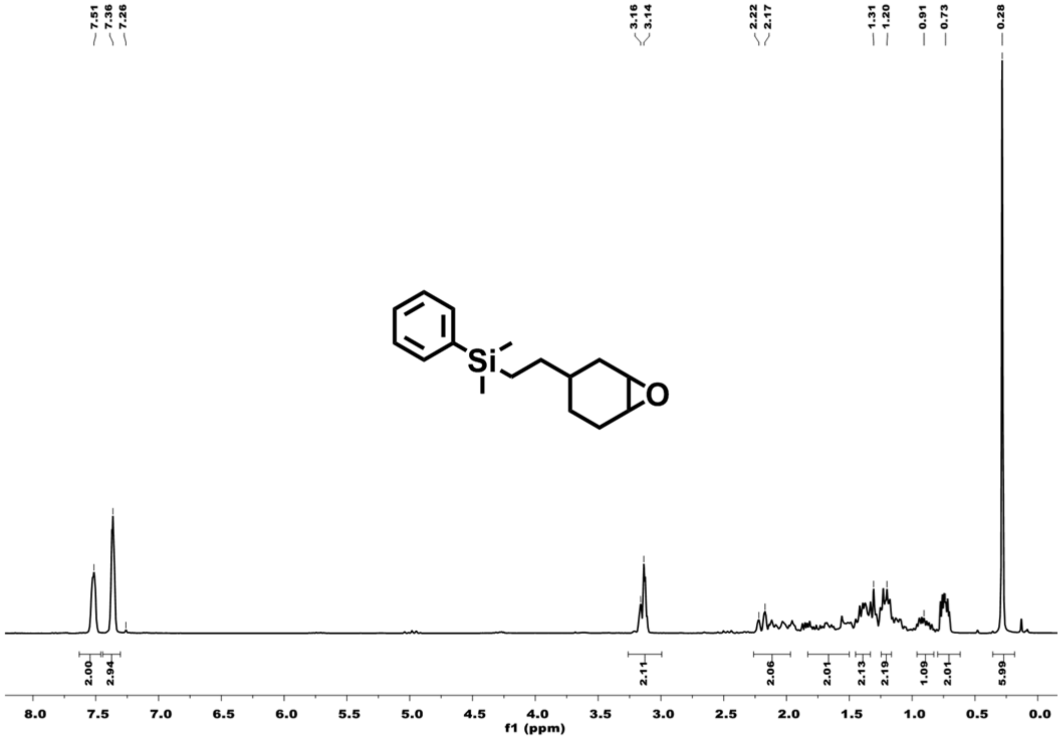

Supplementary Figure 62. ^{1}H-NMR of compound **26**

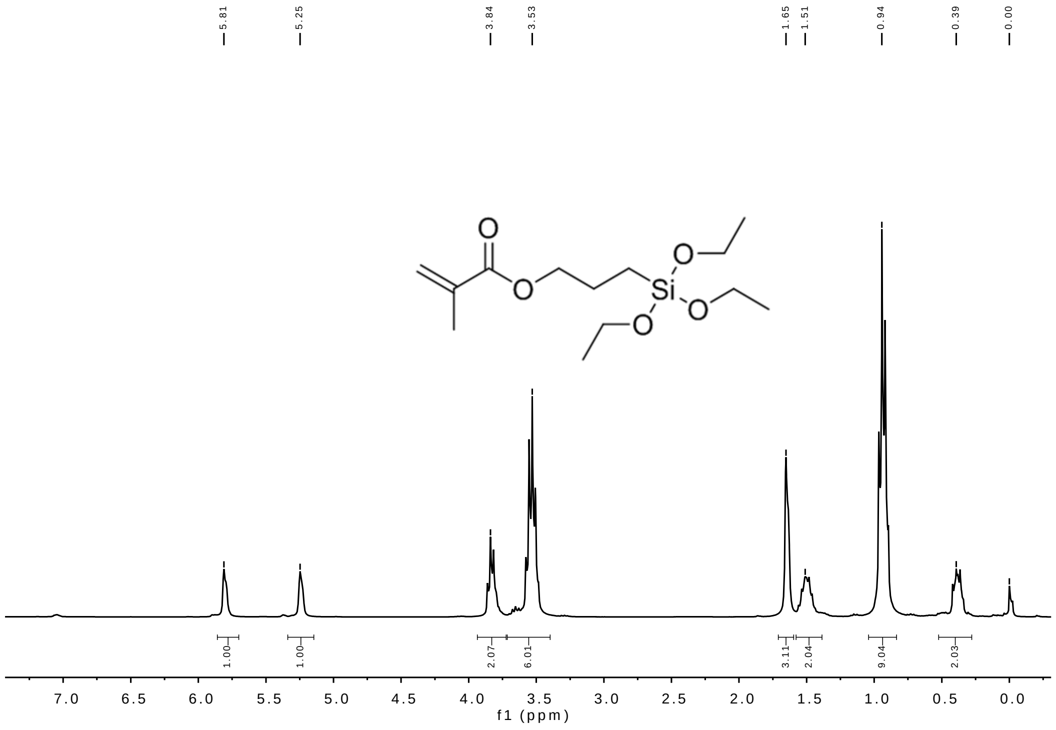

Supplementary Figure 63. ^{1}H-NMR of compound **27**

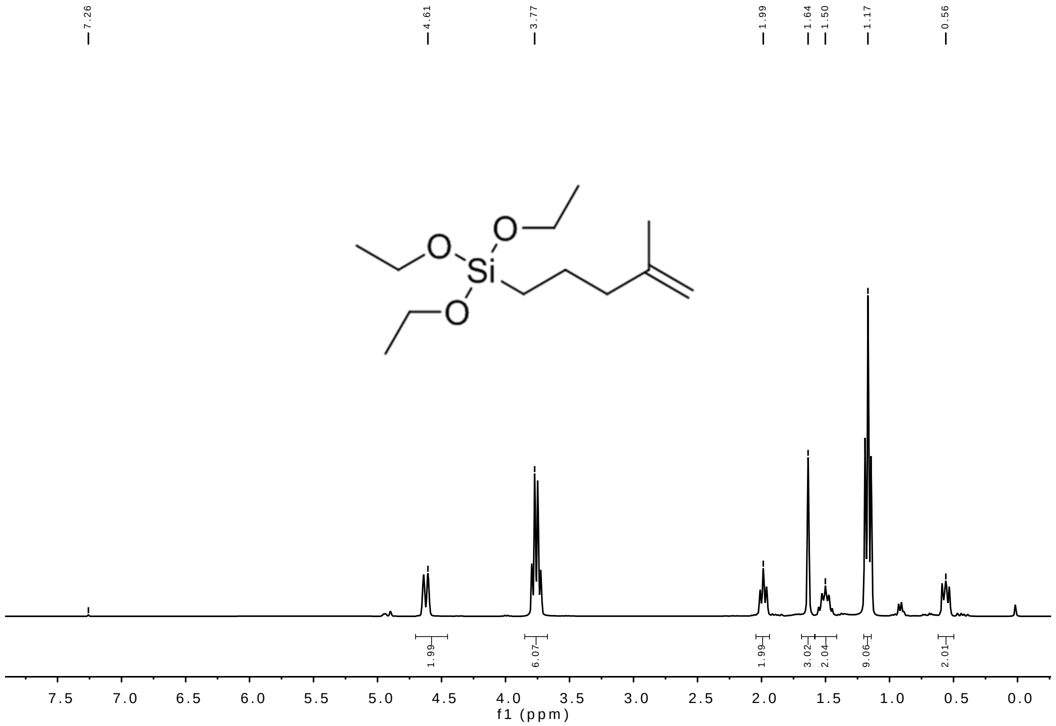

Supplementary Figure 64. ^{1}H-NMR of compound **28**

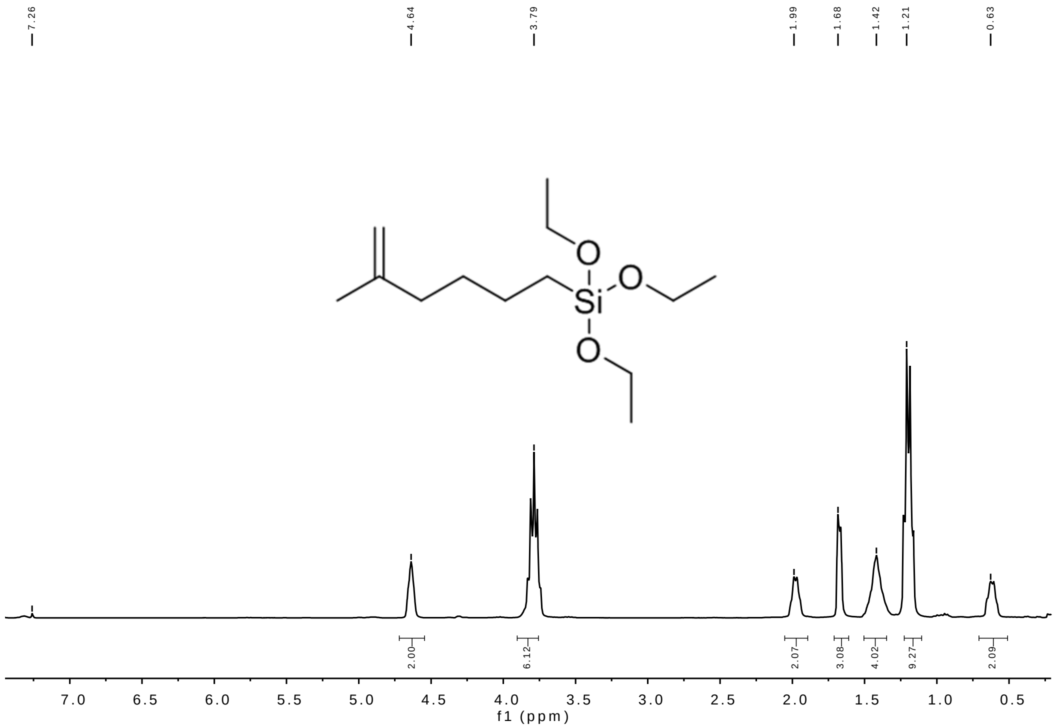

Supplementary Figure 65. ^{1}H-NMR of compound **29**

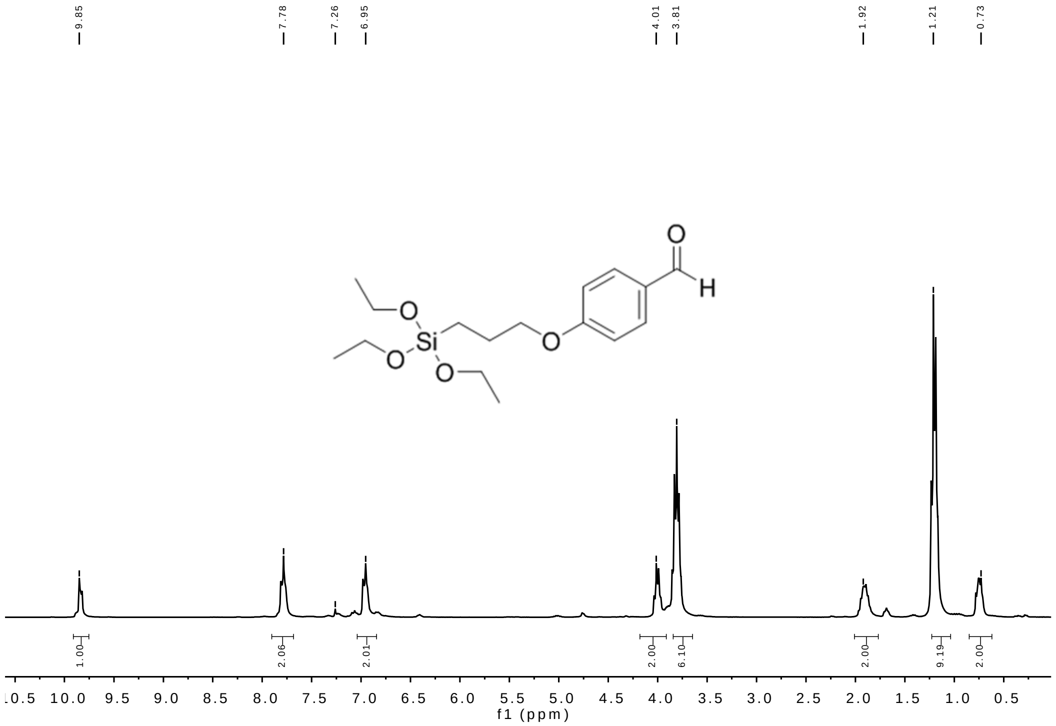

Supplementary Figure 66. ^{1}H-NMR of compound **30**

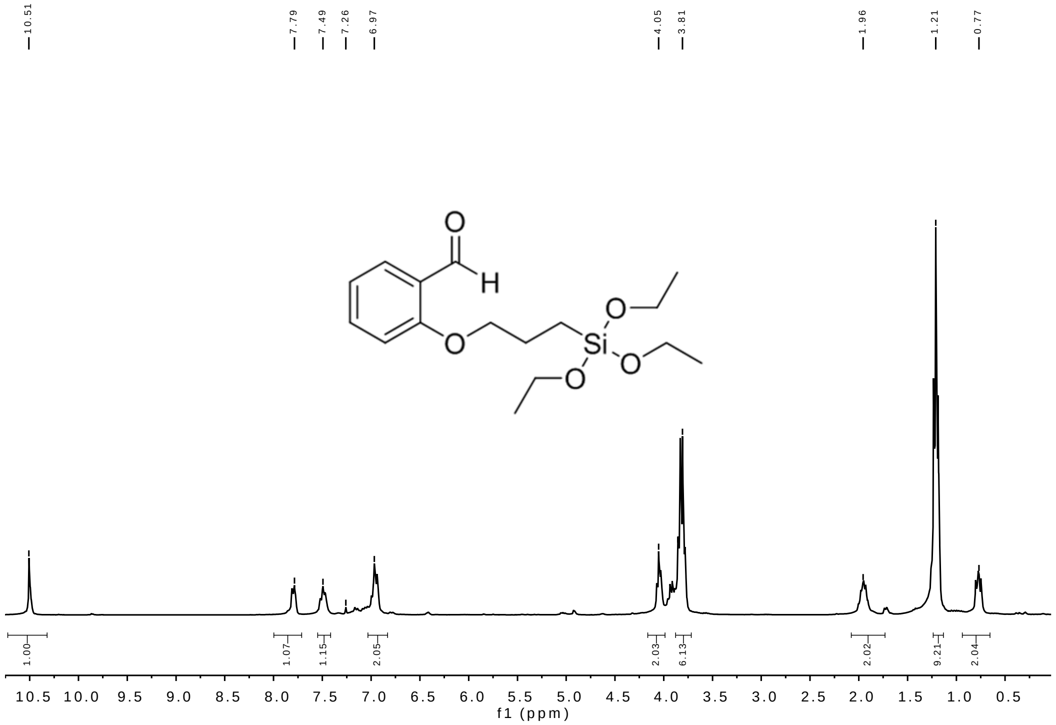

Supplementary Figure 67. ^{1}H-NMR of compound **31**

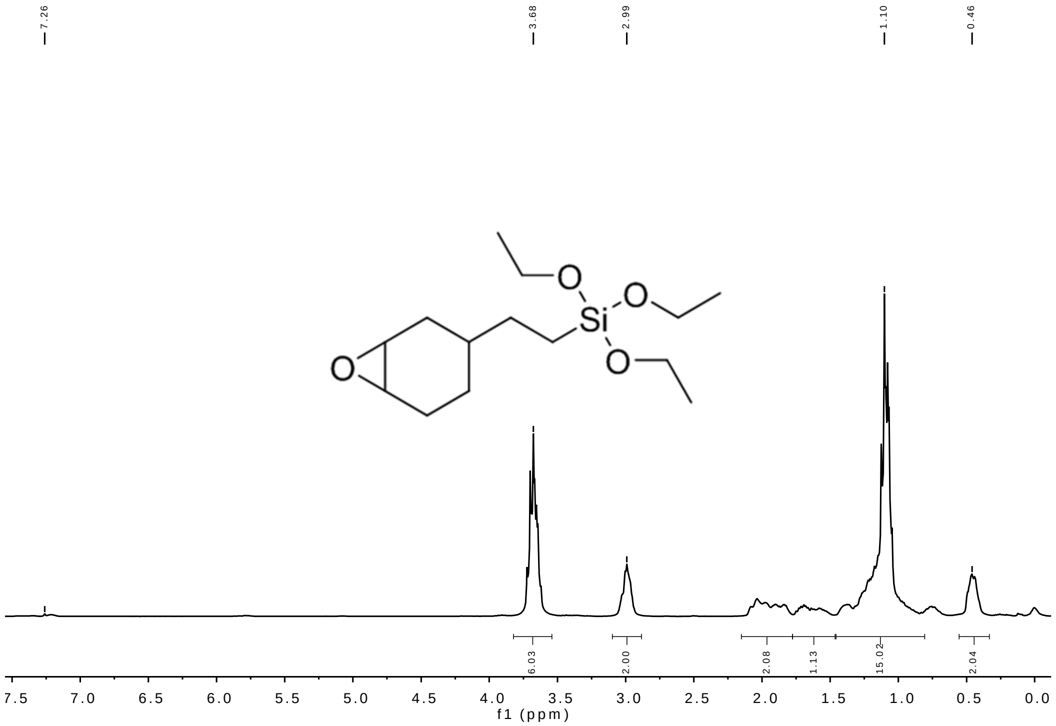

Supplementary Figure 68. ^{1}H-NMR of compound **32**

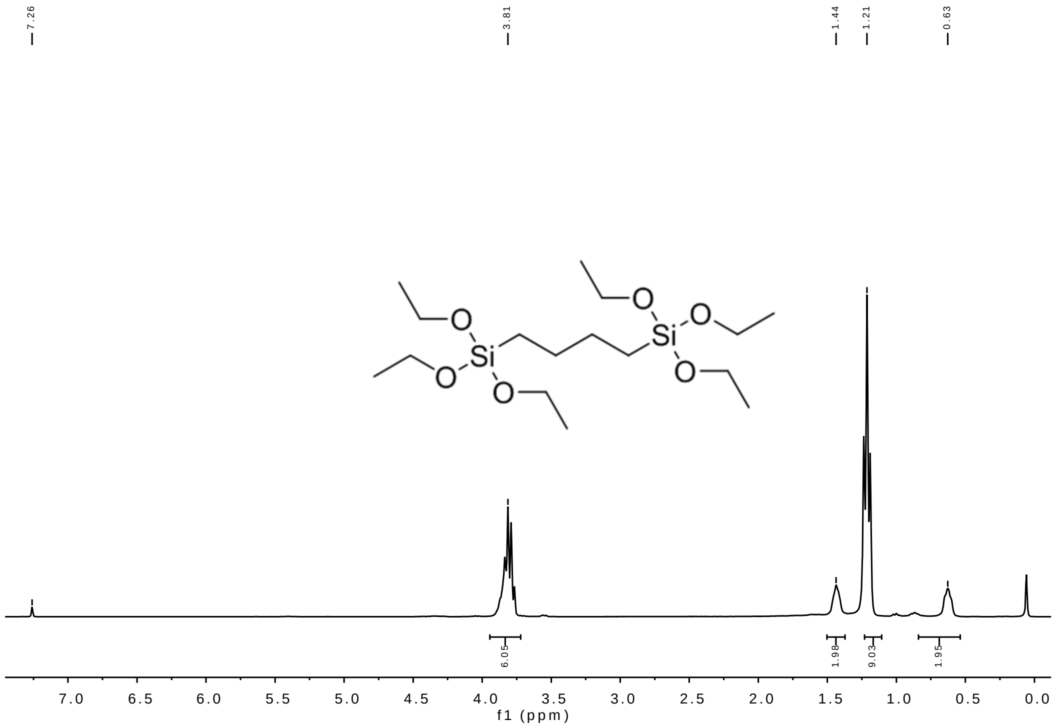

Supplementary Figure 69. ^{1}H-NMR of compound **33**

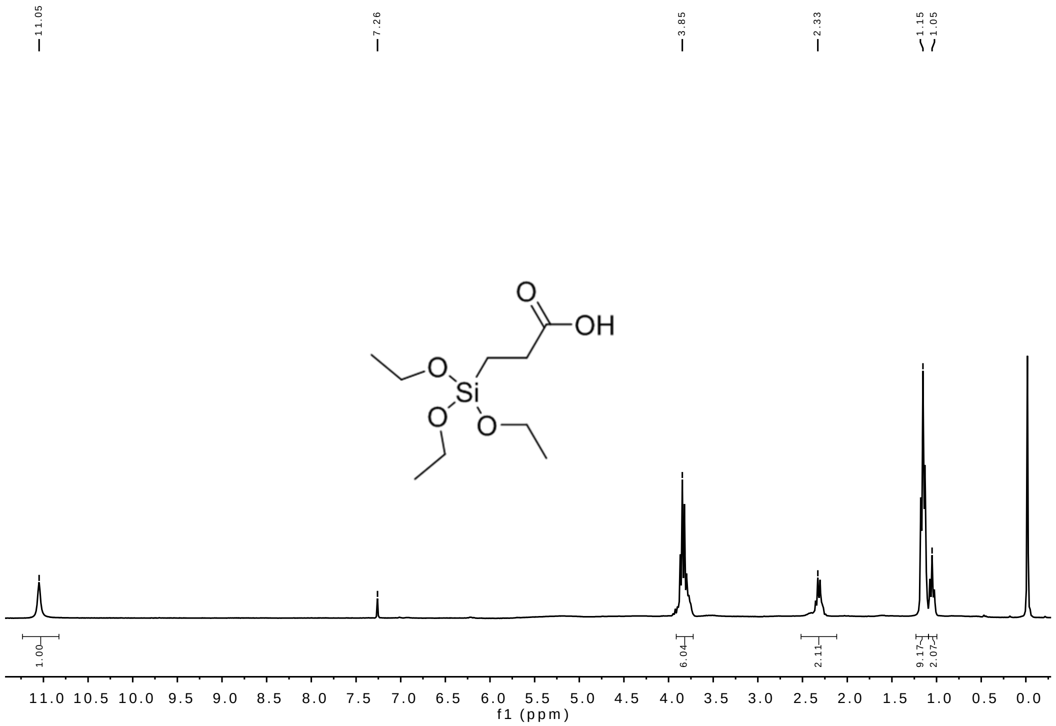

Supplementary Figure 70. ^{1}H-NMR of compound **34**

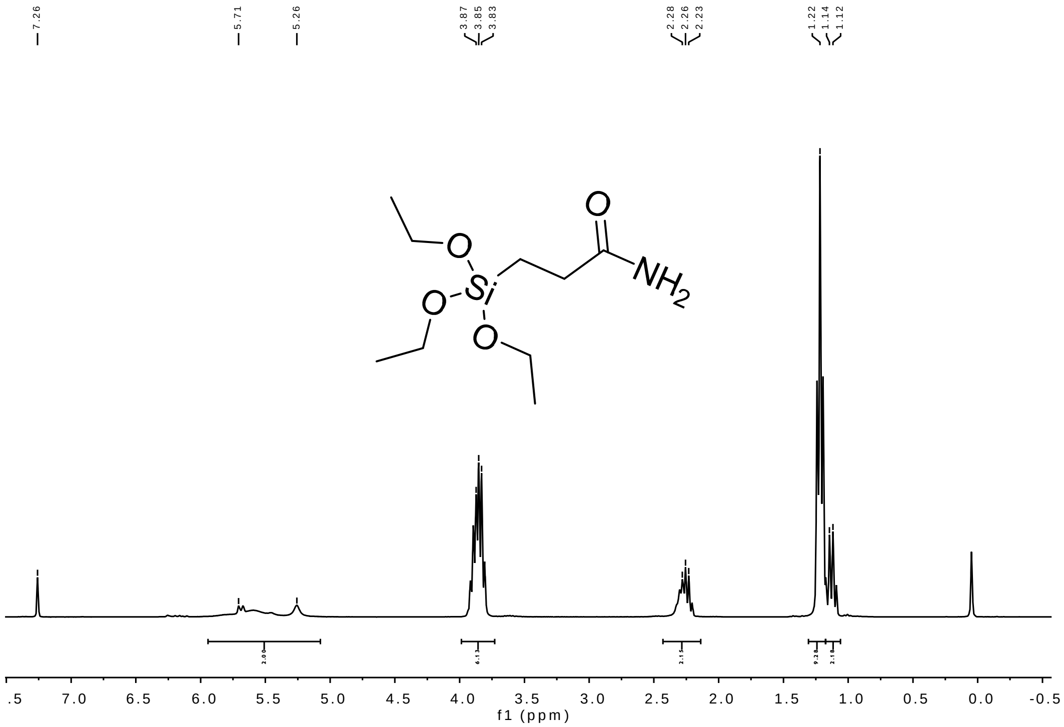

Supplementary Figure 71. ^{1}H-NMR of compound **35**

Supplementary References

1. Tugcu, N., Park, S. K., Moore, J. A. & Cramer, S. M. Synthesis and characterization of high-affinity, low-molecular-mass displacers for anion-exchange chromatography. *Ind. Eng. Chem. Res*. **41**, 6482–6492 (2002).
2. Rathore, R. C. & Burns, L. Preparation of hexakis(4 - Bromophenyl) Benzene (HBB) [1,1′:2′,1″-Terphenyl, 4,4″-dibromo - 3′,4′,5′,6′-tetrakis(4-bromophenyl)-]. *Org. Synth.* **82**, 30–33 (2005).
3. Kojima, T. & Hiraoka, S. Selective alternate derivatization of the hexaphenylbenzene framework through a thermodynamically controlled halogen dance. *Org. Lett.* **16**,1024–1027 (2014).
4. Furukawa, H. et al. Ultrahigh porosity in metal-organic frameworks. *Science* **329**, 424–428 (2010).
5. Hiraoka, S., Yamauchi, Y. Arakane, R. &Shionoya, M. Template-directed synthesis of a covalent organic capsule based on a 3 nm-sized metallocapsule. *J. Am. Chem. Soc.* **131**, 11646−11647 (2009).
6. Prakash, M. J. et al. Metal−organic polyhedron based on a CuII paddle-wheel secondary building unit at the truncated octahedron corners. *Inorg. Chem.* **48**, 1281−1283 (2009).
7. Willems, T. F., Rycroft, C. H., Kazi, M., Meza, J. C. & Haranczyk, M. Algorithms and tools for high-throughput geometry-based analysis of crystalline porous materials. *Microporous and Mesoporous Materials* **149**, 134−141 (2012).
8. Wang, S. et al. Ultrafine Pt nanoclusters confined in a calixarene-based {Ni24} coordination cage for high-efficient hydrogen evolution reaction. *J. Am. Chem. Soc.* **138**, 16236−16239 (2016).
9. Albano, V. G., Natile, G. & Panunzi, A. Five-coordinate alkene complexes of palladium (II) and platinum (II). *Coordin. Chem. Rev.* **133**, 67−114 (1994)
10. Ahn, C.C.& Krivanek, O.L. *EELS Atlas: A Reference Guide of Electron Energy Loss Spectra Covering All Stable Elements,* Gatan Inc., Warrendale, 1983.
11. Chen, Y., Ji, S., Sun, W., Chen, W., Dong, J., Wen, J., Zhang, J., Li, Z., Zheng, L., Chen, C., Peng, Q., Wang, D. & Li, Y. Discovering partially charged single-Atom Pt for enhanced anti-Markovnikov alkene hydrosilylation. *J. Am. Chem. Soc.* **140**, 7407−7410 (2018)
