# Supplementary material for: Biomimetic caged platinum catalyst for hydrosilylation reaction with high site selectivity
Source: Nat Commun. 2021 Jan 4;12:64. doi: 10.1038/s41467-020-20233-w (PMC7782696; doi:10.1038/s41467-020-20233-w)
Supplement: Supplementary file 3 — Description of Additional Supplementary Files [file 41467_2020_20233_MOESM3_ESM.pdf]

Description of Additional Supplementary Files

File Name: Supplementary Movie 1
Description: The rotating structural model of **MOP1**

File Name: Supplementary Movie 2
Description: The rotating structural model of **COP1-T-Pt**.
